# Long-term activity of tandem CD19/CD20 CAR therapy in refractory/relapsed B-cell lymphoma: a single-arm, phase 1–2 trial

**DOI:** 10.1038/s41375-021-01345-8

**Published:** 2021-07-16

**Authors:** Yajing Zhang, Yao Wang, Yang Liu, Chuan Tong, Chunmeng Wang, Yelei Guo, Dongdong Ti, Qingming Yang, Shen Qiao, Zhiqiang Wu, Weidong Han

**Affiliations:** 1 Department of Bio-therapeutic, the First Medical Center, Chinese PLA General Hospital, Beijing, China; 2 Medical Big Data Research Centre, Medical Innovation Research Division, Chinese PLA General Hospital, Beijing, China

**Keywords:** Phase II trials, Cancer immunotherapy

## Abstract

Increasing the remission rate and reducing the recurrence rate can improve the clinical efficacy of chimeric antigen receptor (CAR) T cell therapy in recurrent/refractory non-Hodgkin lymphoma (r/rNHL). In this open-label, single-arm phase I/II trial, 87 patients with r/rNHL, including 58 patients with aggressive diffuse large B-cell lymphoma and 24 with high tumour burden, received an infusion at doses of 0.5 × 10^6^–8 × 10^6^ TanCAR7 T cells per kilogram of body weight after conditioning chemotherapy. The best overall response rate was 78% (95% confidence interval [CI], 68–86); response rates were consistent across prognostic subgroups. The median follow-up was 27.7 months. The median progression-free survival was 27.6 months (95% CI, 11 to not reached). Cytokine release syndrome (CRS) occurred in 61 patients (70%) with 60% of cases being grade 1 or 2 and 10% being grade 3 or greater. Grade 3 CAR T cell-related encephalopathy syndrome (CRES) occurred in 2 patients (2%). Two patients died from treatment-associated severe pulmonary infection, and one died from CRS-related pulmonary injury between 1 and 3 months post infusion. Long-term remissions were observed following the use of TanCAR7 T cells in r/rNHL with a safety profile that included CRS but few cases of CRES.

## Introduction

Non-Hodgkin lymphoma (NHL) is a frequent haematologic malignancy [1]; ~80% of NHL patients can achieve complete remission with traditional treatments [2–5]. However, for patients with recurrent/refractory NHL (r/rNHL), these treatments have limited efficacy, and the percentage of diffuse large B-cell lymphoma (DLBCL) patients who achieve 1-year survival is less than 30% [6–8].

Chimeric antigen receptor (CAR) T cells are engineered T cells that can be induced to express on their surface single-chain antibody fragments that target tumour cell surface antigens [9, 10]. Published clinical data have shown that CAR T cell therapy with CD19 (CD19 CAR T cell therapy) can result in disease remission in 52–82% of patients with r/rNHL [11–16] and even in 93% of patients with mantle-cell lymphoma (MCL) [17]. Although CD19 CAR T cell therapy has resulted in a breakthrough in the treatment of r/rNHL, several new and relevant problems have emerged that urgently need to be resolved [18, 19]. In NHL patients who benefit from CD19 CAR T cell therapy, despite prevalent adverse events (AEs), such as cytokine release syndrome (CRS), the most prominent problem from the perspective of long-term clinical efficacy is recurrence after remission [20–22]. Given the large clinical experience with CD19 CARs, antigen loss is considered to be the main method mediating the recurrence of acute lymphocytic leukaemia patients [23, 24]. Although CD19 expression in NHL has not been systematically studied during relapse after CAR T cell therapy, emerging data provide evidence that this phenomenon also occurs in NHL [12, 15]. Strategies to further improve the efficacy of CAR T cell therapy and reduce the recurrence rate are needed.

Previously, we screened and optimised tandem CD19/CD20 CAR-engineered T cells (TanCAR7 T cells) that can target two antigens simultaneously or separately for NHL treatment. We found that T cells with a TanCAR7 structure possessed robust antitumour activity in vitro, and we showed early evidence of the safety and efficacy of TanCAR7 T cells in r/rNHL treatment [25]. To further confirm the promising results and investigate the key covariates affecting the response rate, relapse rate, and safety of TanCAR7 T cell therapy, the first 28 patients in the interim report were followed up for an extended time and an extension cohort was recruited. Here, we combine these two patient cohorts and present the updated analysis from this study, including the best overall response rate, duration of response, progression-free survival (PFS), overall survival (OS), and AEs.

## Methods

### Study design and participants

This open-label, single-arm phase I/II trial (ClinicalTrials.gov number NCT03097770) was approved by the Ethics Committee of the Chinese PLA General Hospital (Beijing, China), and informed consent was obtained from all patients. The study was conducted from May 2017 through January 2020 at the Biotherapeutic Department of the Chinese PLA General Hospital. The date of data cut-off for the efficacy evaluation was March 2021.

The patient characteristics are shown in Table 1 and Table S1 in the Supplementary Information. All patients were diagnosed with NHL, including 58 with DLBCL (67%), 13 with follicular lymphoma (FL, 15%), 6 with transformed follicular lymphoma (tFL, 7%), 5 with primary mediastinal B-cell lymphoma (PMBCL, 6%), 2 with chronic lymphocytic leukaemia and small lymphocytic lymphoma (2%), 2 with MCL (2%), and 1 with mucosa-associated lymphoid tissue lymphoma (1%), based on the 2008 World Health Organization guidelines. Patients eligible for inclusion in this study had to meet all the key eligibility criteria, including age ≥16 and ≤70 years, Eastern Cooperative Oncology Group (ECOG) score between 0 and 2, histologically confirmed CD20+ and/or CD19+ expression, and refractory disease or relapse after treatment with ≥2 lines of chemoimmunotherapy including an anti-CD20 antibody and alkylating agents. Patients eligible for this study could not meet any of the following criteria: definite involvement of the gastrointestinal tract confirmed by endoscopy, the detection of a clear HAMA effect for prior CD19 CAR T cells, and uncontrolled active bacterial or viral infection. The detailed eligibility and exclusion criteria are provided in the Supplementary Information.

**Table 1:** Baseline characteristics.

Characteristics	No. of patients % (infusion) (*n* = 87)	No. of patients % (leukapheresis) (*n* = 92)
Age (years), no. (%)		
<60	71 (82%)	75 (82%)
≥60	16 (18%)	17 (18%)
Sex, no. (%)		
Male	41 (47%)	42 (46%)
Female	46 (53%)	50 (54%)
ECOG performance status score, no. (%)		
0–1	54 (62%)	59 (64%)
2	33 (38%)	33 (36%)
Disease stage at study entry		
I or II	13 (15%)	14 (15%)
III or IV	74 (85%)	78 (85%)
Diagnosis by central histologic review, no. (%)		
DLBCL	58 (66%)	61 (66%)
tFL	6 (7%)	7 (8%)
FL	13 (15%)	13 (14%)
PMBCL	5 (6%)	5 (5%)
Others	5 (6%)	6 (7%)
IHC		
CD20^**+**^	87 (100%)	92 (100%)
CD19^**+**^	78 (89%)	83 (90%)
CD19^**−**^	4 (5%)	4 (4%)
CD19 missing data	5 (6%)	5 (5%)
Double or triple protein expression: MYC plus BCL2, BCL6 or both, no. (%)		
Yes	30 (34%)	33 (36%)
No	38 (44%)	40 (43%)
Missing data	19 (22%)	19 (21%)
Ki-67 (≥70%)	54 (62%)	57 (62%)
Extranodal organ involvement, no. (%)		
Yes	62 (71%)	65 (71%)
No	25 (29%)	27 (29%)
No. of previous lines of antineoplastic therapy, no. (%)		
≤2	23 (27%)	24 (26%)
3–5	49 (56%)	52 (57%)
≥6	15 (17%)	16 (17%)
Refractory or relapse, no. (%)		
Refractory	70 (80%)	74 (80%)
Relapse to second-line or later therapy	17 (20%)	18 (20%)
Relapse after ASCT	12 (14%)	12 (13%)
Relapse after previous CD19 CAR T cell therapy	9 (10%)	9 (10%)
Tumour burden		
SPD ≥ 100 cm^**2**^	24 (28%)	27 (29%)
SPD < 100 cm^**2**^	63 (72%)	65 (71%)
Bulky/non-bulky disease		
Lesion diameter ≥ 10 cm	19 (22%)	21 (23%)
Lesion diameter < 10 cm	68 (78%)	71 (77%)

Advanced stage disease was defined as stage III or IV according to the modified Ann Arbor staging system with higher stage numbers indicating greater dissemination of cancer through the body [40].

ECOG performance status values range from 0 to 5 with higher scores indicating increasing disability.

The sum of the product of the diameters (SPD) was calculated as the sum of the long axis and short axis of all measurable sites.

Relapse to ASCT

• Disease progression or relapse ≤12 months after ASCT (biopsy-proven recurrence required for relapsed subjects).

Relapse after previous CD19 CAR T cell therapy

• Disease progression or relapse ≤6 months after previous CD19 CAR T cell therapy (biopsy-proven recurrence required for relapsed subjects).

*ASCT* autologous stem cell transplantation, *CAR* chimeric antigen receptor, *DLBCL* diffuse large B-cell lymphoma, *ECOG* Eastern Cooperative Oncology Group, *FL* follicular lymphoma, *IHC* immunohistochemistry, *PMBCL* primary mediastinal B-cell lymphoma, *SPD* sum of the product of the diameters, *tFL* transformed follicular lymphoma.

### CAR T-cell manufacturing and lymphodepleting chemotherapy

The TanCAR7 construct was generated by linking the CD19 single-chain variable fragment (scFv) derived from the FMC63 monoclonal antibody (mAb) and the CD20 scFv derived from the Leu-16 mAb18 in frame with the hinge and transmembrane domains of CD8 and the cytoplasmic domains of 4-1BB and CD3 zeta. TanCAR7 T cells were manufactured as described previously [25]. After a conditioning regimen of fludarabine and cyclophosphamide (FC; cyclophosphamide (20–30 mg/kg divided into 3 days) and fludarabine (20–30 mg/m^2^ × 3 days)), the first 7 patients received infusions in the range of 0.5–6 × 10^6^ CAR+ cells per kilogram of body weight. Subsequently, an extended dose range of cell infusion was tested in further studies, and 21 r/r NHL patients were treated with TanCAR7 T cells at a target dose of 1–8 × 10^6^ TanCAR7 T cells/kg after receiving a conditioning regimen. To observe the relationship between infusion doses and efficacy, after interim analysis, a target dose of 2–8 × 10^6^ TanCAR7 T cells/kg was selected for subsequent patients. The actual infusion dose for individual patients was mainly determined by the number of CAR T cells that could be obtained after 8–10 days of culture (Supplementary Table 2). All infused cells were freshly manufactured. To reduce the risk of CRS after CAR T cell infusion, we added doxorubicin (10 mg/m^2^ for 1 day) on the basis of the FC dose for patients with high tumour burden (bulky disease, defined as the maximum diameter of a single lesion >10 cm or the sum of the products of the longest perpendicular diameters (SPD) ≥ 100 cm^2^).

### End points and assessments

The primary analysis was conducted when 87 patients could be evaluated 6 months after TanCAR7 T cell infusion. The primary end point was safety (phase I), and the best overall response rate included the percentages of patients achieving a complete response (CR) and a partial response (PR) (phase II) as assessed by the investigators according to the International Working Group Response Criteria for Malignant Lymphoma at 3, 6, 12, 18 ± 3, 24 ± 3, and 36 ± 3 months post infusion [26]. Evaluation was performed every 12 months thereafter. Secondary end points included response duration, PFS, OS, cellular kinetics data and serum cytokine levels for all patients who received an infusion. Details regarding the response criteria are provided in the Supplementary Information. The cell-of-origin subtype, blood level of CAR T cells and serum cytokine levels were assessed as described previously [25].

### Statistical analysis

The probability of occurrence is presented as a percentage. Exact methods (Clopper–Pearson 95% confidence intervals (CIs)) were used for categorical variables. Forest plots include CIs both for individual groups and for the difference between the groups and the reference group. The time of the first CAR T cell infusion was used as the origin in the time-to-event analyses of PFS and OS. PFS and OS were estimated using the Kaplan–Meier method and were compared with the use of the log-rank test. The 2-sided 95% CIs for the median duration of response, PFS, and OS were calculated using STATA version 15.0 software. Differences between two categories with respect to continuous parameters were determined using an exact Wilcoxon rank sum test.

## Results

### Patient characteristics

**Fig. 1:**
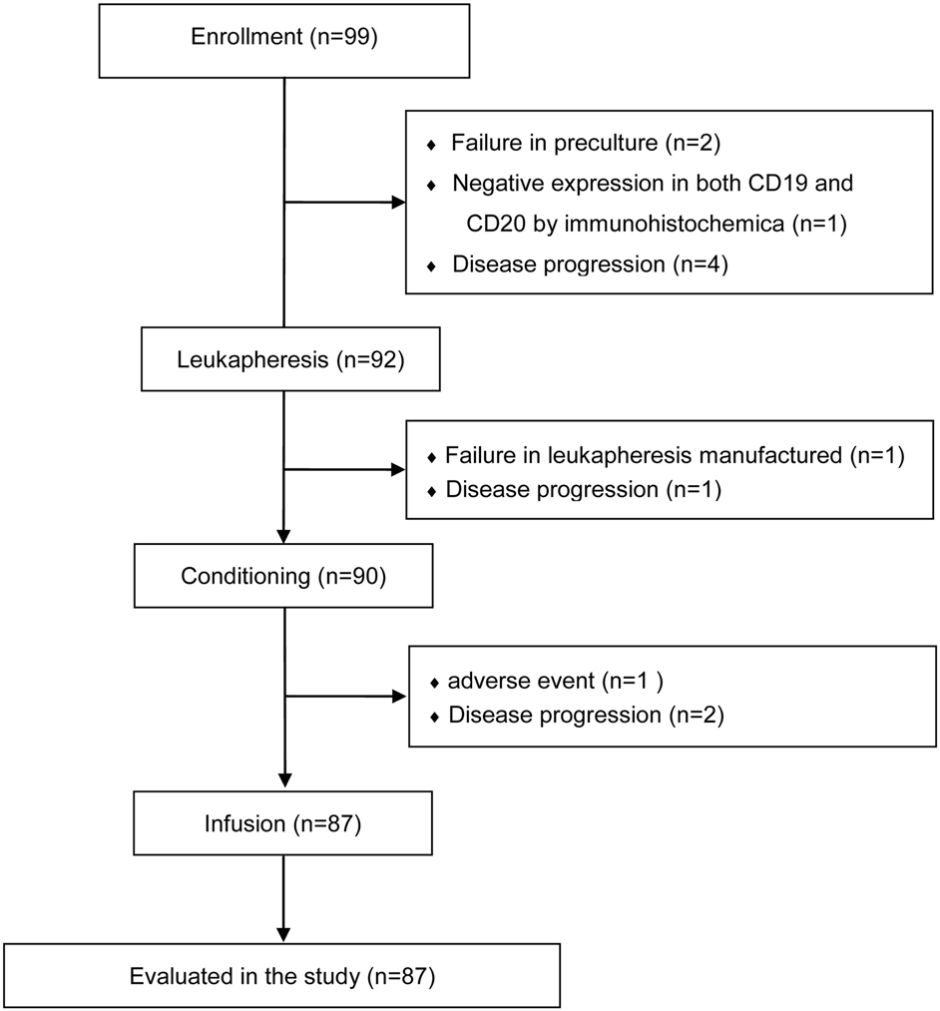
CONSORT flow diagram. Among the 7 patients who were excluded from the study during screening, 2 failed in preculture, 1 had negative expression of both CD19 and CD20 by immunohistochemistry, and 4 had disease progression while waiting for hospitalisation. Among the 92 enroled patients, one patient failed leukapheresis, and another had AEs after leukapheresis. Before infusion, 1 patient had serious AEs, and 2 could not tolerate pretreatment due to disease progression. AEs adverse events.

From May 2017 to January 2020, a total of 99 patients were enroled, and leukapheresis was performed in 92 patients. Leukapheresis failed in one patient, and another patient experienced disease progression after leukapheresis. Before infusion, 1 patient had serious AEs, and 2 patients could not tolerate lymphodepleting chemotherapy due to disease progression resulting in secondary hepatic and renal failure. Manufactured TanCAR7 T cells were administered to 87 patients (Fig. 1). Among the patients receiving TanCAR7 T cell infusion, the median age was 50 years (range, 17–68). Most of the patients (85%) had stage III or IV disease, 38% had an ECOG performance status score of 2, 62% were positive for Ki-67 in more than 70% of cells in a given biopsy, 71% had extranodal organ involvement, 73% had received at least three previous therapies, 80% had refractory disease, 14% experienced relapse after transplantation, 28% had high disease burden, and 22% had bulky disease. Among the aggressive B-cell lymphoma patients, 8 of 29 (28%) patients had double-/triple-hit status. We also included nine patients (10%) who experienced relapse after anti-CD19 CAR T cell infusion (Table 1).

All of the patients with prior CD19 CAR T therapy had positive expression of both CD19 and CD20 as confirmed by immunohistochemistry prior to TanCAR7 therapy, and also had a recovery of B cells. CD19 CAR T cells from the prior therapy were not detectable at the time of enrolment.

### Outcomes

**Fig. 2:**
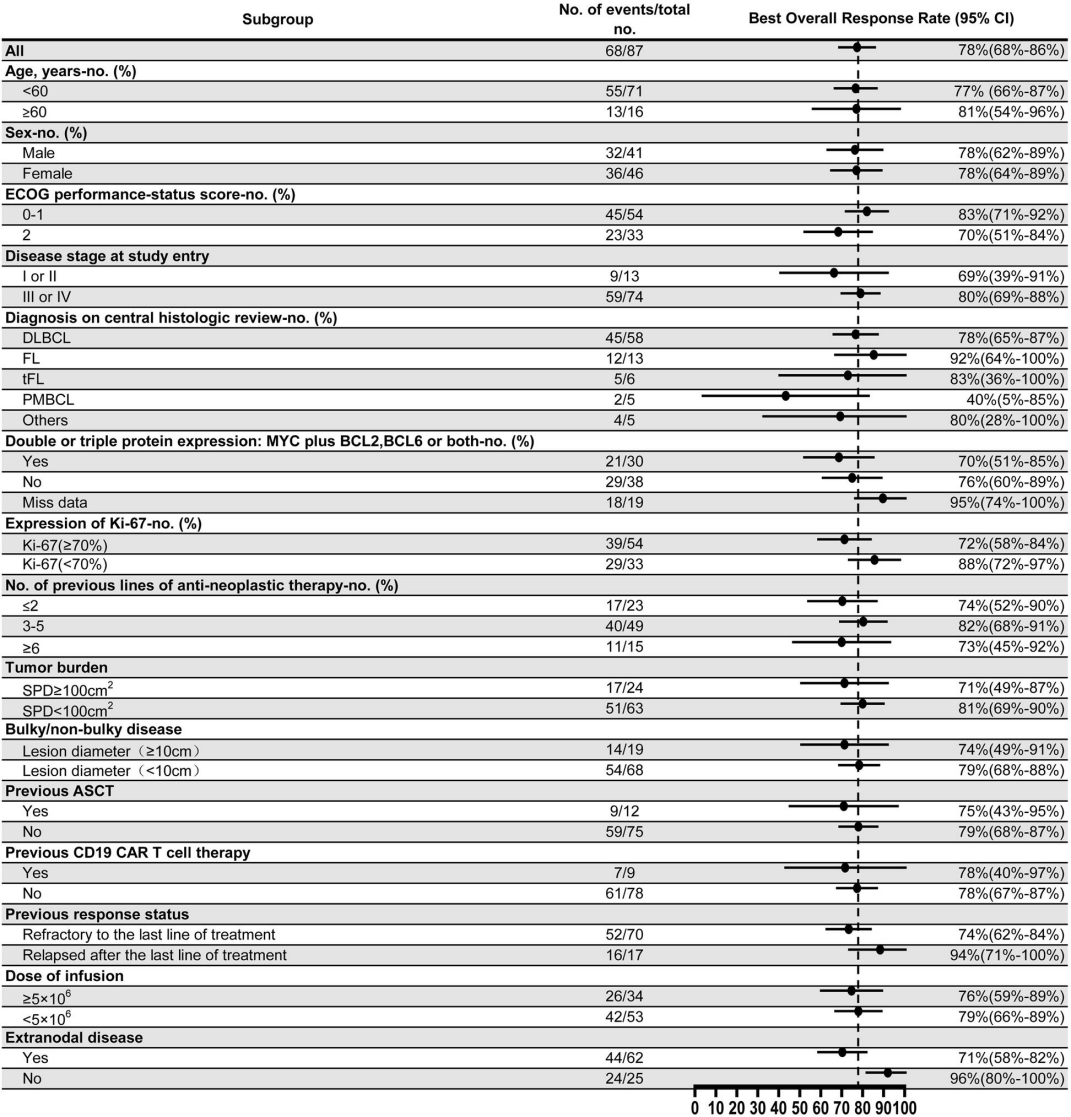
Best overall response rate based on subgroup. ASCT autologous stem cell transplantation, CAR chimeric antigen receptor, CI confidence interval, DLBCL diffuse large B-cell lymphoma, ECOG Eastern Cooperative Oncology Group, FL follicular lymphoma, PMBCL primary mediastinal B-cell lymphoma, SPD sum of the product of the diameters, tFL transformed follicular lymphoma.

All patients who received TanCAR7 T cell infusion were included in the efficacy evaluation. The data cut-off date for the efficacy evaluation was March 2021. Among a total of 87 patients, the best overall response rate was 78% (95% CI, 68–86) with 70% (95% CI, 59–79) of the patients achieving a CR and 8% (95% CI, 3–16) experiencing a PR (Table 2, Supplementary Fig. 1). Among the patients with an objective response, 66 (97%) obtained the best overall response when evaluated at 3 months, and 2 (3%) with a PR at 3 months after infusion improved to a CR at 5 months. Among patients with aggressive B-cell lymphomas, including DLBCL, tFL and PMBCL, 52 of 69 had a response (75%; 95% CI, 64–85), and 47 of 69 achieved a CR (68%; 95% CI, 56–79). Among patients with FL, 12 of 13 had a response (92%; 95% CI, 64–100), and 10 of 13 achieved a CR (77%; 95% CI, 46–95). Among patients with DLBCL, 45 of 58 exhibited a response (78%; 95% CI, 65–87). The occurrence of an overall response did not differ significantly according to prognostic subgroups, including groups classified according to age, sex, ECOG performance status score, Ki-67 expression, clinical stage, tumour burden and infusion dose. Responses (78%; 95% CI, 40–97) were also consistent in patients who had progressive disease (PD) during their most recent line of therapy and in those who received CD19 CAR T cell therapy (Fig. 2). In an intent-to-treat analysis that included all 92 enroled patients, the overall response rate was 74% (95% CI, 64–83) (Table 2).

**Table 2:** Response characteristics.

	Efficacy (95% CI)
Best overall response (all, *n* = 87)	78% (68–86%)
Complete response	70% (59–79%)
Partial response	8% (3–16%)
Progressive disease	16% (9–26%)
Could not be evaluated	6% (2–13%)
Ongoing response	61% (44–75%)
Best overall response (aggressive:DLBCL, PMBCL and tFL, *n* = 69)	75% (64–85%)
Complete response	68% (56–79%)
Partial response	7% (2–16%)
Progressive disease	19% (10–30%)
Could not be evaluated	6% (2–14%)
Best overall response (DLBCL, *n* = 58)	78% (65–87%)
Complete response	71% (57–82%)
Partial response	7% (2–17%)
Progressive disease	21% (11–33%)
Could not be evaluated	2% (0–9%)
Best overall response (FL, *n* = 13)	92% (64–100%)
Complete response	77% (46–95%)
Partial response	15% (2–45%)
Progressive disease	8% (0–36%)
Could not be evaluated	0
Response of intent-to-treat (*n* = 92)	74% (64–83%)
Median duration of response, months (95% CI)	NR (23.4-NE)
Median progression-free survival, months (95% CI)	27.6 (11-NE)
Median overall survival, months (95% CI)	NR (36.7-NE)

All patients who received TanCAR7 T cell infusion were included in the efficacy evaluation. The best overall response rate was calculated as the percentage of patients who had a complete and partial response.

*CI* confidence interval, *DLBCL* diffuse large B-cell lymphoma, *FL* follicular lymphoma, *NE* could not be estimated, *NR* not reached, *PMBCL* primary mediastinal B-cell lymphoma, *tFL* transformed follicular lymphoma.

During a median follow-up of 27.7 months, 60% of the patients continued to be in remission. At the time of the data cut-off, 45 patients including 7 patients with high tumour burden, remained in remission, and four patients either started alternative therapy before disease progression (*n* = 3) or were lost to follow-up (*n* = 1). Thirty-four patients experienced disease progression, including 14 patients with primarily resistant disease, 16 with PD after a CR and 4 with PD after a PR following TanCAR7 T cell infusion (Supplementary Figs. 1 and 2). Of the relapsed patients, thirty had new lesions, but 9/13 had no progression of their primary lesion. The duration of response appeared to be influenced by the ECOG performance-status score, disease stage at enrolment, tumour burden, and extranodal disease (Supplementary Table 3). However, the limited number of patients investigated did not provide the statistical power needed for correlative analysis. Among the 16 relapsed patients, which represents less than 30% of patients with a CR, twelve patients were successfully biopsied. Three patients either died before being biopsied or failed to be biopsied due to lesions close to vital organs; 1 patient was biopsied but no viable tumour tissue was obtained since he was previously treated with a BTK inhibitor and glucocorticoid in another hospital. The results from biopsy analysis and transgene copy number detection showed that 7 (58%) patients did not have detectable CAR T cells in either PB or tumour biopsies, 4 (33%) were being examined for potential causes, and one (8%) patient had a loss of CD19/CD20 dual antigens. No patients lost CD19 only or CD20 only. The antigen-negative recurrence rate was far lower than that found in the CD19-CAR T cell study [21].

**Fig. 3:**
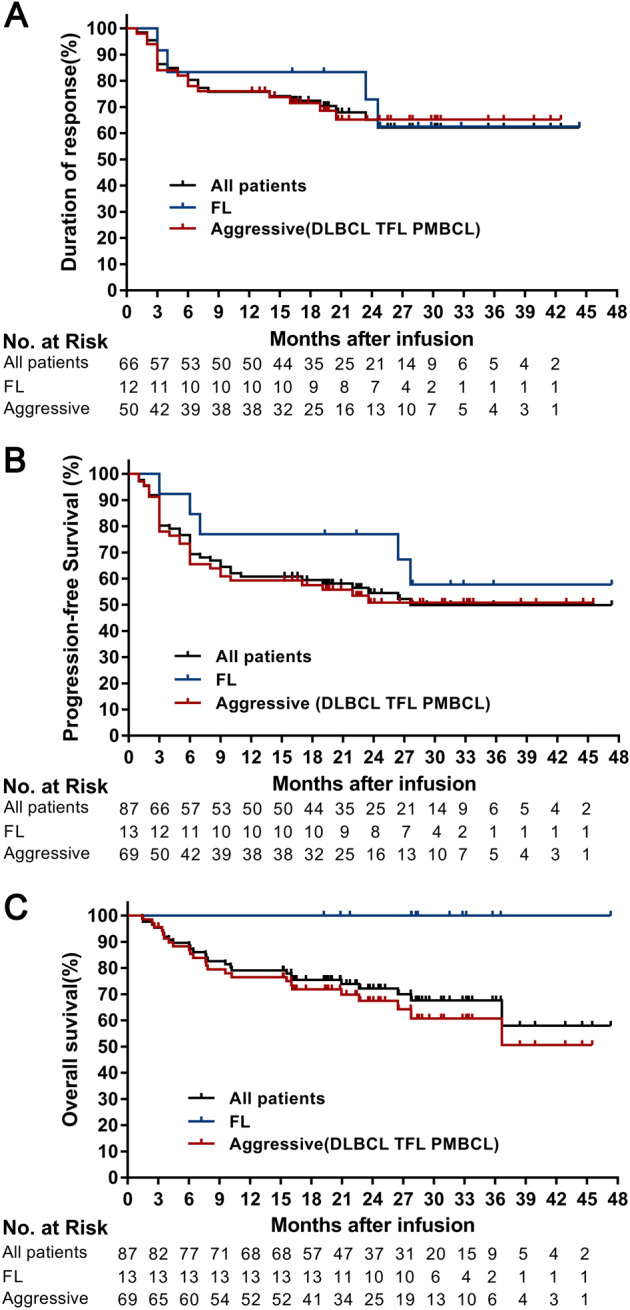
Kaplan–Meier estimates of the duration of response, progression-free survival, and overall survival. **A** Shows the duration of response of the 66 patients who had a response, including the patients with aggressive disease (including DLBCL, tFL and PMBCL) or FL. **B** Shows progression-free survival for all 87 patients who received an infusion, including the patients with aggressive disease (including DLBCL, tFL and PMBCL) or FL. The progression-free survival time was recorded as the date of TanCAR7 T cell infusion to the date of disease progression or death from any cause. Subjects not meeting the criteria for progression by the data analysis cut-off date were censored at their last evaluable disease assessment date. Data from 4 other patients were censored: 3 received interventional treatment, and 1 was lost to follow-up. **C** Shows the overall survival for all 87 patients who received an infusion or patients with aggressive disease (including DLBCL, tFL and PMBCL) or FL. The overall survival time was recorded as the date of TanCAR7 T cell infusion to the date of death from any cause. Patients who did not have an event had their data censored for the analyses at the date at which they were last known to be alive. DLBCL diffuse large B-cell lymphoma, FL follicular lymphoma, PMBCL primary mediastinal B-cell lymphoma, tFL transformed follicular lymphoma.

The median duration of response was not reached (95% CI, 20.9 to not reached), with 80% (95% CI, 69–88) of the patients remaining in remission at 6 months and 76% (95% CI, 64–84) remaining in remission at 12 months (Fig. 3A). Seventy-six percent (95% CI, 62–86) of patients with aggressive B-cell lymphomas and 83% (95% CI, 48–96) of patients with FL maintained the response at 12 months (Fig. 3A). The median PFS was 27.6 months (95% CI, 11 months to not reached) with PFS rates of 69% (95% CI, 58–78) at 6 months and 61% (95% CI, 50–70) at 12 months (Fig. 3B). Among patients with aggressive B-cell lymphomas, the median PFS was 23.9 months (95% CI, 9 to not reached), and 59% (95% CI, 46–70) of these patients were progression-free at 12 months. Among patients with FL, the median PFS was not reached, and 77% (95% CI, 44–92) were progression-free at 12 months (Fig. 3B). Even in the patients with SPD ≥ 100 cm^2^, the median PFS was 6 months (95% CI, 6 months to not reached), with a PFS rate of 49% (95% CI, 27–68) at 6 months and 44% (95% CI, 23–64) at 12 months (Supplementary Fig. 3). PFS was not affected by the dose of infusion (Supplementary Fig. 4). The median OS was not reached with OS rates of 90% (95% CI, 81–94) at 6 months and 79% (95% CI, 69–86) at 12 months (Fig. 3C). The estimated probability of survival at 12 months was 77% (95% CI, 65–85) among patients with aggressive B-cell lymphomas and 100% (95% CI, NE to NE) of patients with FL (Fig. 3C). A total of 74% of patients who received an infusion remained alive at the time of the data cut-off.

Among patients with DLBCL, the CR rate was 71% (95% CI, 57–82), and the median duration of response was not reached (95% CI, 16 to not reached) with 74% (95% CI, 59–85) of the patients who exhibited a response remaining in remission 12 months after having a response (Supplementary Fig. 5A). The median PFS was 23.5 months (95% CI, 9 months to not reached), and 59% (95% CI, 45–71) showed no disease progression at 12 months after infusion (Supplementary Fig. 5B). No significant difference in PFS was found between patients with germinal centre‐derived B-cell lymphoma and those with non-germinal centre‐derived B-cell lymphoma(Supplementary Fig. 5C). The median OS was not reached, and the estimated probability of survival was 88% (95% CI, 76–94) at 6 months and 75% (95% CI, 62–86) at 12 months (Supplementary Fig. 5D).

Nine patients had previously received CD19-CAR T cell treatment. Seven patients had a response, with 6 patients achieving a CR and 1 experiencing a PR. Four patients who had a response still maintained the response at the time of the data cut-off. Three patients who had achieved complete remission relapsed at 6 months, 20.5 months and 24.6 months. No significant differences in DOR were noted between those patients and the patients with no history of anti-CD19 CAR T cell infusion (Supplementary Fig. 6). The results of the analysis of biopsies from the 3 patients showed that no patient had a loss of CD19 antigens at relapse.

### Safety

All 87 patients experienced AEs, with 70 (80%) experiencing grade 3 or higher events (Supplementary Table 4). The most common grade 3 or higher events observed within 1 month after infusion included leukopenia (76%), thrombocytopenia (37%) and pyrexia (33%). A total of 61 patients (70%) had CRS, which was grade 1 in 39 patients (45%), grade 2 in 13 patients (15%), grade 3 in 8 patients (9%) and grade 4 in 1 patient (1%). The median time after infusion to the onset of CRS was 1 day (range, 1–9), and the median CRS duration was 6 days (range, 1–11). The median time to the onset of grade 3 or higher CRS was 1 day (range, 1–9). Among patients with grade 2 or higher CRS, 15 received monotherapy or combined therapy with tocilizumab, infliximab, etanercept, and glucocorticoids. The proportion of patients who had CRS was positively associated with the dose of infusion and especially with the tumour burden and CAR T cell expansion. All patients with robust CAR T cell expansion reaching >500 CAR T cells in 1 mL of blood developed CRS (Supplementary Fig. 7). Among the cytokines detected, IL-2, IL-6, TNF-α and CRP were elevated in the patients with CRS (Supplementary Fig. 8). CAR T cell-related encephalopathy syndrome (CRES) occurred in 15 patients (17%) within 4 weeks after infusion (grade 1, *n* = 11; grade 2, *n* = 2; grade 3, *n* = 2). The median time to onset was 7 days (range, 3–17), and the median duration was 12 days. The most common grade 1 or 2 neurologic events were tremor (9%), anxiety (8%) and a disturbance in attention (3%). The only grade 3 neurologic event was seizures (*n* = 2). No relationship between the infusion dose and the grade of CRES was apparent (Supplementary Fig. 9). For the two patients with seizures, levetiracetam was given to prevent secondary seizures, and for the other13 patients with suspected grade 1–2 early neurologic signs, levetiracetam was given for sseizure prevention. No new cases of CRS or CRES related to TanCAR7 T cells were reported after 1 month. Our analyses suggested that additional doxorubicin conditioning can increase the degree of bone marrow suppression but has no significant effect on bone marrow recovery (Supplementary Fig. 10). Transient B-cell depletion occurred in all 68 patients who had a response, and 19/65 (29%) patients had sustained normal CD19 + B-cell recovery at ~6 months (Supplementary Fig. 11).

Three patients died from treatment-associated complications. One patient died from severe pulmonary infection associated with persistent unrecoverable myelosuppression at 1.5 months. The second patient with bulky disease (SPD = 156 cm^2^) was evaluated as achieving a PR at 1 month but died from CRS-related pulmonary injury. The third patient with multiorgan involvement (SPD = 130 cm^2^) was assessed as achieving a PR at 1 month but died from severe pulmonary infection with secondary multiple organ failure 2 months after infusion.

### CAR expansion and persistence

The peak expansion of TanCAR7 T cells occurred within the first 7–14 days post infusion, and the median peak number of circulating CAR T cells was 168.95/μl blood (range, 3–4786.96). Blood CAR T cell levels were higher in patients who achieved a response (PR or better) than in those who did not achieve a response, as measured by the maximum vector transgene copies per microgram of genomic DNA, maximum absolute number of CAR T cells and area under the curve during the first 28 days after the infusion. TanCAR7 T cells were detectable in the blood (by means of qPCR analysis) for up to 400 days in 30 patients with ongoing CR (Supplementary Fig. 12). To determine the impact of persistence on relapse, we also analysed blood CAR T cell levels at 21–40 and 41–60 days after CAR T cell infusion. CAR T cell levels at both 21–40 and 41–60 days after CAR T-cell infusion were not significantly different between patients who had an ongoing response and those who had relapsed at the time of the data cut-off (Supplementary Fig. 12). The results of biopsy analysis and transgene copy number detection in 12 patients at the time of relapse showed that 5 patients still had detectable CAR T cells in tumour biopsies.

## Discussion

To reduce the rate of disease recurrence due to antigen loss after single-target CAR T cell infusion, many strategies have emerged from the perspective of increasing antigen coverage [20, 27–31]. Previously, we found that TanCAR7 T cells, in which two scFvs with different antigen specificities were connected to a single molecule, not only showed broad antigen coverage, including both CD19 and CD20, but also possessed robust antitumour activity in vitro [25]. Here, to further confirm the promising results and to study the key covariates affecting the response and duration of TanCAR7 T cells, we report an updated analysis of TanCAR7 T cells in a larger patient population with r/rNHL. In this study, all patients were characterised as having relapsed or refractory disease, and some patients had an extremely high tumour burden or aggressive disease. However, impressive responses were achieved, and the results of the efficacy analysis showed no significant difference between all 87 patients and the first 28 patients in the interim report. The best overall response rate was 78%, and the CR rate was 70%. Remarkably, the median PFS was not reached until 27.6 months; 80% of patients remained alive at 12 months, and the recurrence rate in patients with CR was only 10% at 6 months after infusion. In addition, the generation of a clinical response was rapid in terms of the earlier occurrence time of CRS with a median time to the onset of CRS of 1 day (range, 1–9) after infusion.

The results of subgroup analyses showed that high response rates (≥70%) were also observed in patients with aggressive disease, refractory disease, or extranodal disease at baseline. Among patients with DLBCL, the best overall response rate was 78% (95% CI, 65–87); 70% of patients experienced a CR. Although the overall response rate tended to be lower in patients with PMBCL, patients with extramediastinal lesions benefitted significantly, and we consider that the difference may be related to the special microenvironment of PMBCL [32]. It is gratifying that CR was still achieved in patients with disease that was refractory to or failed CD19-CART treatment, and the response duration was similar to that in patients with no history of CD19-CART treatment, suggesting that TanCAR7 T cell therapy may be used in the future as a new treatment option for patients who fail CD19-CART treatment. The previously reported trials showed a median PFS of ~6 months following CD19-CAR T cell treatment in r/rNHL [12, 13, 15]. In our study, disease progression did not predominantly occur early as it did with CD19-CAR T cells. Instead, a large proportion of PD cases occurred after the initial 6 months, suggesting that more patients who benefitted in this trial achieved longer remission. In addition, we observed that relapses were more likely to occur in patients with a high tumour burden, extranodal disease, and stage III or IV disease. Although robust CAR T cell expansion is associated with high rates of CR, given the presence of not only a more complex microenvironment but also more diverse tumour cell clones in larger lesions [33, 34], appropriate combination therapy strategies may be needed to improve the sustained clinical efficacy in NHL patients with large target volumes.

The expansion of TanCAR7 T cells was strong. Peak expansion was observed within the first 7–14 days post infusion, and the expansion and persistence were greater in patients who had a response than in those who did not have a response. This finding is similar to those of other studies of CAR T cell therapy [12, 13]. There was no significant difference in the persistence of CAR T cells between patients who relapsed and patients with sustained response. In addition, of 12 relapsed patients who underwent biopsies, only 1 had antigen-negative relapse. Therefore, the cause of tumour relapse after TanCAR7 T cells needs additional exploration. Given the differences in trial designs, patient populations and costimulatory molecules selected, it was challenging to compare the results from TanCAR7 T cell therapy to those from single-targeted CAR T cell therapy. The results from anti-CD19 CAR T cell therapy with CD28 showed that permanent persistence of CAR T cells is not required for the maintenance of remission in patients with lymphoma [35]. The long-term persistence of CTL019 cells was observed in 50% patients who achieved complete remissions beyond 1 year [36]. In this trial, TanCAR7 T cells were detectable in 60% of patients with ongoing CR for up to 400 days by means of qPCR analysis. Antigen loss is one of the important reasons for relapse after CD19-CAR T cell treatment [21, 37]. In other reported clinical studies, the antigen-negative recurrence rate of NHL after CAR T cell treatment is ~8–33% [36, 38]. The antigen-negative recurrence rate in this study was only 8%, which suggested that TanCAR7 T cell therapy may reduce the chance of antigen-negative recurrence. Given that progressive CRS can cause serious morbidity in patients or reduce the clinical benefit due to the use of measures intended to control CRS, such as corticosteroids, CAR T therapy with a low incidence of CRS may not only alleviate toxicity but also improve the likelihood of therapeutic benefit [39]. In this trial, CRS was mostly grade 1 or 2 with 10% of cases being grade 3 or higher. Patients with grade 2 or higher CRS were treated with monotherapy or a combination therapy of tocilizumab, infliximab, etanercept, and glucocorticoids. Such treatment did not appear to negatively affect CAR T cell expansion or the treatment response. Finally, the safety profile of TanCAR7 T cells was assessed at doses as high as 8.0 × 10^6^ CAR^+^ T cells.

In conclusion, TanCAR7 T cells showed a potent and durable antitumour response in a heavily pretreated population of patients with NHL.

## Supplementary information

SUPPLEMENTAL MATERIAL
