# Supplementary material for: Long-term activity of tandem CD19/CD20 CAR therapy in refractory/relapsed B-cell lymphoma: a single-arm, phase 1–2 trial
Source: Leukemia. 2021 Jul 16;36(1):189–96. doi: 10.1038/s41375-021-01345-8 (PMC8727291; doi:10.1038/s41375-021-01345-8)
Supplement: Supplementary file 1 — SUPPLEMENTAL MATERIAL [file 41375_2021_1345_MOESM1_ESM.docx]

**Supplementary Information**

**Contents**

**Supplementary Methods**

**Figure S1-S12**

**Table S1-S4**

**Subject inclusion criteria**

Patients eligible for inclusion in this study had to meet all of the following criteria:

1. Age ≥16 and ≤70 years.

2. Eastern Cooperative Oncology Group (ECOG) performance status score between 0 and 2.

3. Histologically confirmed CD20+ and/or CD19+ B-cell non-Hodgkin lymphoma (NHL), including the following types defined by the World Health Organization (WHO) 2008:

• Diffuse large B-cell lymphoma (DLBCL) not otherwise specified.

• Primary mediastinal large B-cell lymphoma (PMBCL).

• Transformed follicular lymphoma (tFL).

• FL.

• Some indolent lymphomas including mantle-cell lymphoma (MCL) and chronic lymphocytic leukaemia/small lymphocytic lymphoma (CLL/SLL).

• Mucosa associated lymphoid tissue (MALT).

4. Refractory disease or relapse after treatment with ≥2 lines of chemotherapy, including rituximab and anthracycline and either having failed autologous hematopoietic stem cell transplantation (HSCT), being ineligible for autologous HSCT or not consenting to autologous HSCT.

We defined chemotherapy-refractory disease as meeting one or more of the following criteria:

• No response to first-line therapy (primary refractory disease).

• No response to second-line or later therapy.

• Progressive disease (PD) as the best response to the most recent therapy regimen.

• Stable disease (SD) as the best response after at least 2 cycles of the most recent line of therapy with an SD duration of no longer than 6 months from the last dose of therapy.

Failure following autologous HSCT was defined as follows:

• PD or relapsed disease ≤12 months after autologous stem cell transplantation (ASCT) (requires biopsy-proven recurrence in relapsed subjects).

• No response or relapse after salvage therapy is given post-ASCT.

5. PD or relapse ≥3 months after treatment with targeted CD19 therapy, including CD19 CAR T cells or anti-CD19/anti-CD3.

6. Successful leukapheresis assessment and preculture of T cells.

7. Life expectancy > 3 months.

8. Adequate organ function:

• Creatinine < 1.6 mg/dL (140 µmol/L) or creatinine clearance ≥60 mL/min.

• Alanine aminotransferase (ALT)/aspartate aminotransferase (AST) < 3× upper limit of the normal range.

• Bilirubin <2.0 mg/dL unless the subject had Gilbert’s syndrome (<3.0 mg/dL).

• A minimum level of pulmonary reserve defined as ≤ grade 1 dyspnoea and pulse oxygenation > 91% with room air.

• Cardiac ejection fraction ≥50%, no evidence of pericardial effusion as determined by an echocardiogram (ECHO), and no clinically significant electrocardiogram (ECG) findings.

9. An adequate bone marrow reserve defined as:

• Absolute neutrophil count (ANC)>1,000/mm3.

• Absolute lymphocyte count (ALC)≥300/mm3.

• Platelet count ≥ 50,000/mm3.

• Haemoglobin > 7.0 mg/dL.

10. Measurable or assessable disease according to the “IWG Response Criteria for Malignant Lymphoma” (Cheson 2007). Patients in complete remission (CR) with no evidence of disease were not eligible.

11. Informed consent/assent requiring that all patients have the ability to understand and the willingness to provide written informed consent.

**Subject exclusion criteria**

Patients eligible for this study must not meet any of the following criteria:

1. Patients with definite involvement of the gastrointestinal tract. Endoscopy should be performed to confirm gastrointestinal involvement in suspected patients. However, patients with central nervous system (CNS) involvement were cautiously enrolled in this clinical study.

2. Detection of a clear HAMA effect in patients with prior CD19 CAR T cell treatment failure or recurrence, or negative tumour puncture detection of CD19 and CD20.

3. Pregnant or lactating women.

4. Uncontrolled active bacterial, viral infection (active hepatitis B or hepatitis C infection, HIV infection, EBV infection, rubella virus, and so on) or other pathogens (treponema pallidum infection, and so on).

5. Class III/IV cardiovascular disability according to the New York Heart Association Classification and a cardiac ejection fraction ≥50%.

6. History of allogeneic stem cell transplantation.

7. Requirement for urgent therapy due to tumour mass effects such as respiratory obstruction or blood vessel compression.

8. Current or expected need for systemic corticosteroid therapy.

9. Any organ failure.

10. Patients with a second tumour requiring therapy or intervention.

11. Subjects considered unlikely to complete all protocol-required study visits or procedures, including follow-up visits, or comply with the study requirements for participation according to the investigator’s judgement.

**Efficacy assessments**

The primary analysis of efficacy assessments will be based on the recommendations by the International Malignant Lymphomas Imaging Working Group (Cheson Response Criteria and The Lugano Classification 2014).

A patient evaluated to have a response of CR must show no evidence of bone marrow aspirate/biopsy by morphology or by immunohistochemistry (if the bone marrow was involved by lymphoma at baseline), and the spleen and liver must be normal in size with no lymphoma-related B-symptoms in addition to radiological CR.

• A patient will have a best overall disease response of CR if they had standard assessments showing the complete disappearance of all detectable clinical and radiographic evidence of disease and the disappearance of all disease-related symptoms at least 12 weeks after infusion.

• A patient will have a best overall disease response of partial response (PR) if there is a >50% decrease in the SPD of the six largest dominant nodes or nodal masses and no increase in the size of the other nodes, liver or spleen (and the patient would not qualify for CR).

• SD is defined as less than PR (see above) but is not PD (see below) when at least one SD assessment is available 4 weeks after TanCAR7 T cell infusion. PET should be positive in typically FDG-avid lymphoma.

• A patient will be evaluated as having PD if there is a >50% increase from nadir in the SPD of any previously identified abnormal node or if the appearance of any new lesion that is >1.5 cm by radiologic evaluation was observed within 12 weeks after infusion.

• If a patient does not qualify for CR, PR, SD or PD, then their disease response will be not evaluated (NE).

The objective response rate (ORR) is defined as the proportion of patients with either CR or PR per the Lugano Classification (Cheson et al, 2014) as the overall disease response while on study.

The best ORR is defined as the incidence of CR and PR recorded from TanCAR7 T cell infusion until PD or the start of new antitumour therapy, whichever comes first.

**Evaluations of AEs**

All events with an assigned CTCAE grade used the grading scales in the current National Cancer Institute CTCAE version 4.0.

**Sample size**

Published clinical data have shown that CAR T cell therapy with CD19 (CD19 CAR T cell therapy) can result in disease remission in 52-82% of patients with r/rNHL^1-5^. Compared to the objective response rate of 52%, the minimum sample size is 69 patients, which in the primary analysis will provide 87% power to demonstrate statistical significance at the α=0.05 level of significance using a superiority test, if the underlying ORR of TanCAR7 T cells reached 65%. Assuming that approximately 20% of enrolled patients may be lost to follow-up for different reasons, at least 83 patients need to be enrolled to ensure that 69 patients are evaluated.

**Detection of HAMA**

Patient serum samples will be collected prior to entering the group for analysis of HAMA to assess the immunogenicity of CAR T cells. HAMA was detected by ELISA Kit (SBJ-H1543, SenBeiJia Biological Technology) according to the manufacturer’s instructions.

**CAR gene transduction efficiency and immunogenicity**

CAR T cells or peripheral blood samples were analysed by flow cytometry analysis, which was performed as described previously^6^.

**Measurement of serum cytokine levels**

Patient serum was harvested at the following time points: day 1 (prior to TanCAR7 T cell infusion), day 7, day 14, day 21 and day 28. Serum concentrations of TNF-α, IL-2, IL-6, IL-8, IL-10 and C-reactive protein (CRP) were evaluated by ELISA according to the manufacturer’s instructions.

**Transgene copy number quantification by qPCR**

qPCR was carried out by using the 7500 Fast Real-Time PCR system (Applied Biosystems, USA) according to the manufacturer's instructions. Each sample was run in triplicate. A recombinant plasmid containing the transgene was used as a standard for qPCR. The concentration of the purified plasmid DNA was determined by a NanoDrop 2000 spectrophotometer (Thermo, USA). The plasmid copy number in solution was derived from its molar concentration and the Avogadro constant. The copy number gain of the target in the sample was determined by using the comparative Ct (threshold cycle) method, which substitutes the Ct value from the sample with that of the standard curve and then divides it by double the DNA concentration of the sample.

**Statistical analysis**

The population with evaluable response was defined as all patients who received TanCAR7 T cell infusion. Confidence intervals were calculated with the use of the Clopper–Pearson method.

The duration of response (DOR) was defined as the date of the first objective response (which was subsequently confirmed) to disease progression per the revised IWG Response Criteria for Malignant Lymphoma (Cheson 2014) or death regardless of cause. Subjects not meeting the criteria for progression or death by the analysis data cutoff date were censored at their last evaluable disease assessment date, and their response will be noted as ongoing.

Progression-free survival (PFS) was defined as the time from the infusion date to the date of disease progression per the revised IWG Response Criteria for Malignant Lymphoma (Cheson 2014) or death from any cause. Subjects not meeting the criteria for progression by the analysis data cutoff date were censored at their last evaluable disease assessment date.

Overall survival (OS) was defined as the time from infusion to the date of death. Subjects who did not die by the analysis data cutoff date were censored at their last contact date.

**Figure S1. Response to TanCAR7 T cell infusion**

All responses were confirmed and assessed according to the recommendations of the International Conference on Malignant Lymphomas Imaging Working Group. Three patients received new cancer therapy while obtaining PR. One patient lost to follow-up. Patient 3 died from severe pneumonia outside our institute after achieving CR 10 months post infusion, patients 18 and 68 died from a severe pulmonary infection associated with persistent unrecoverable myelosuppression, and patient 44 died from CRS-related pulmonary injury. Eight patients had new lesions in the context of controlled primary lesions. The patients who could not be evaluated are indicated as NE. CR, complete response; NE, could not be evaluated; PD, progressive disease; PR, partial response.


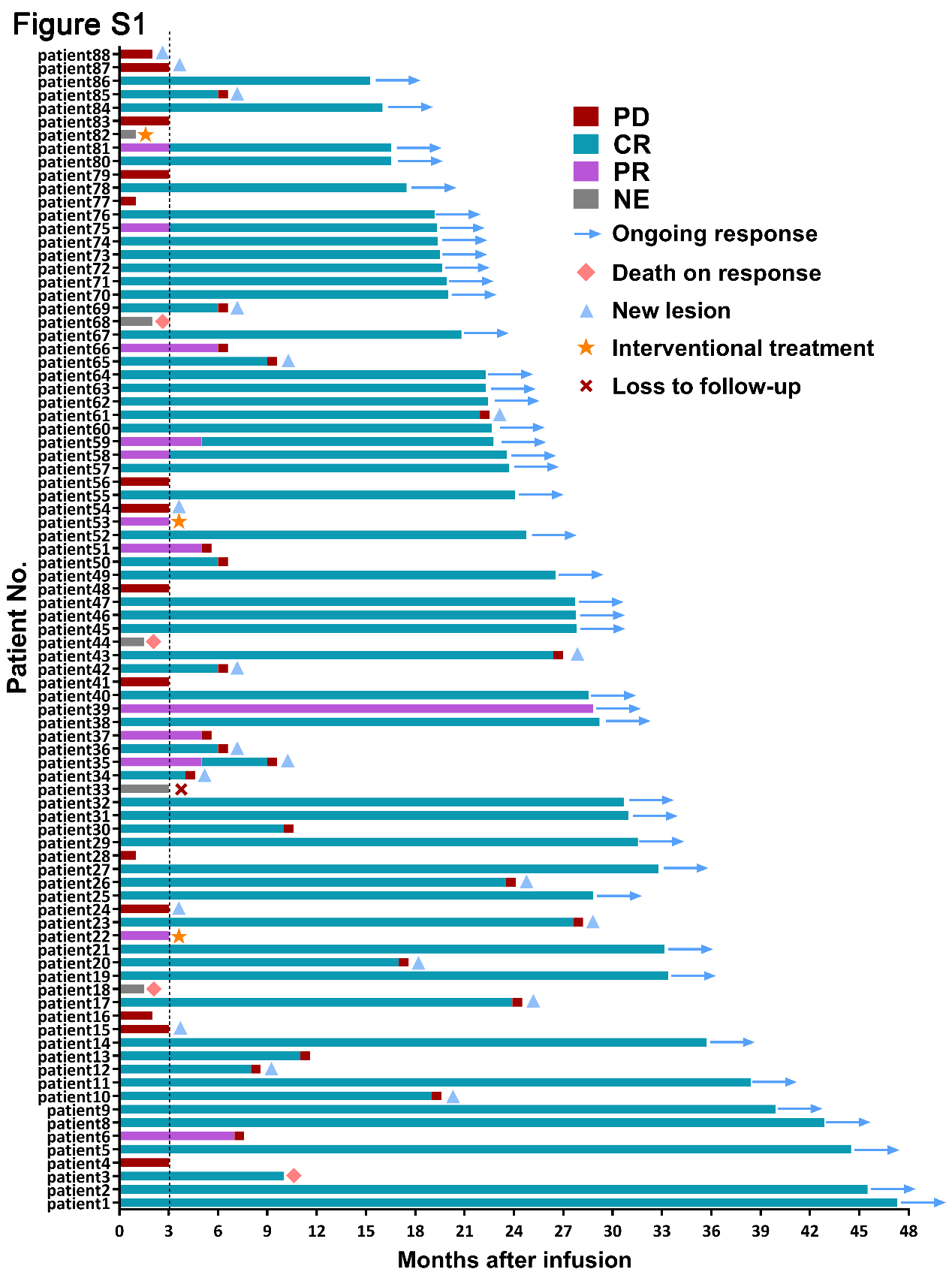


**Figure S2. PET/CT or CT scans of evaluated patients with high tumour burden**

PET/CT or CT scans were performed before and after TanCAR7 T cell therapy. At the time of data cutoff, theses 7 patients with high tumour burden remained in remission. CT, computerized tomography; PET, positron emission tomography.


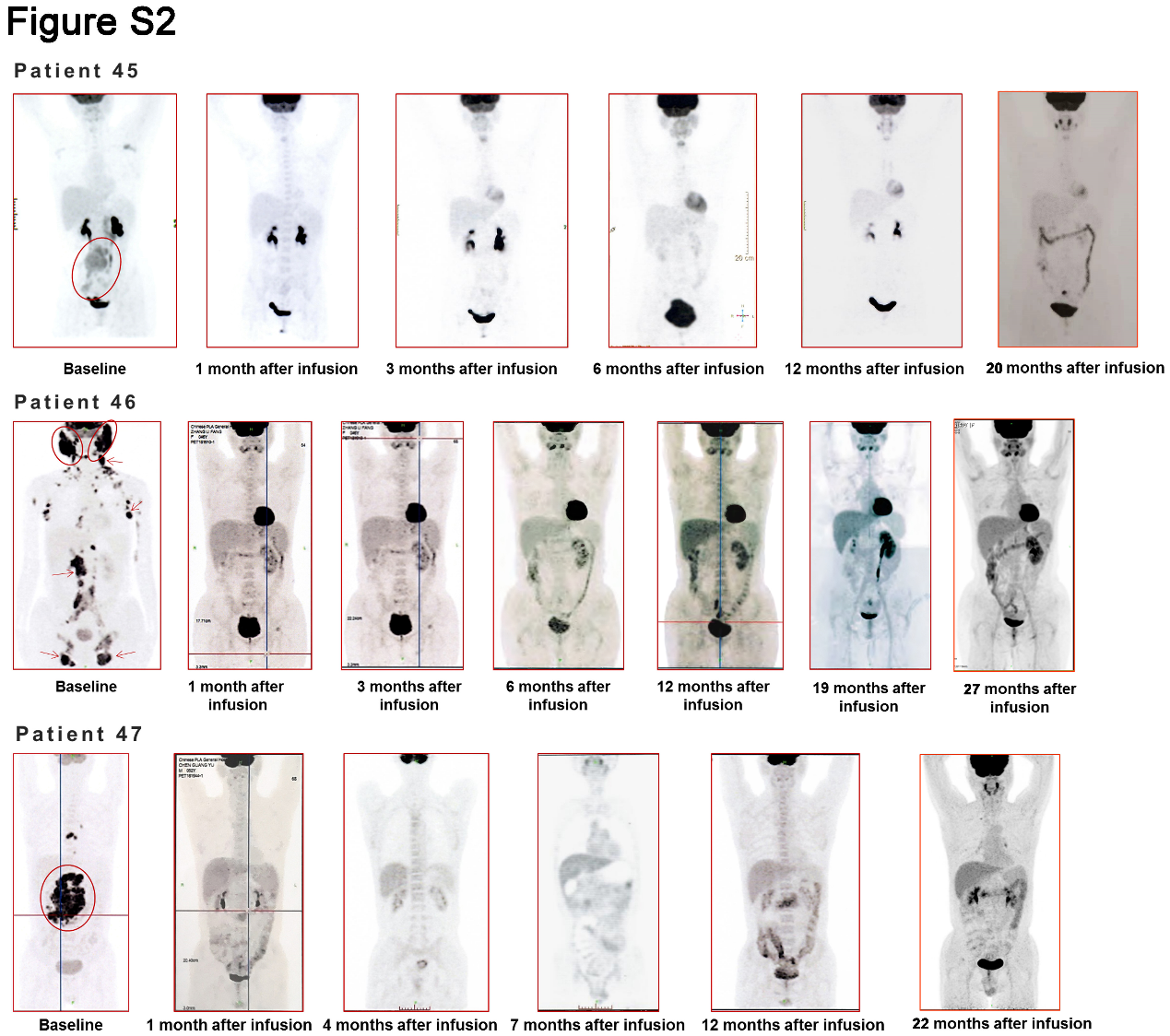


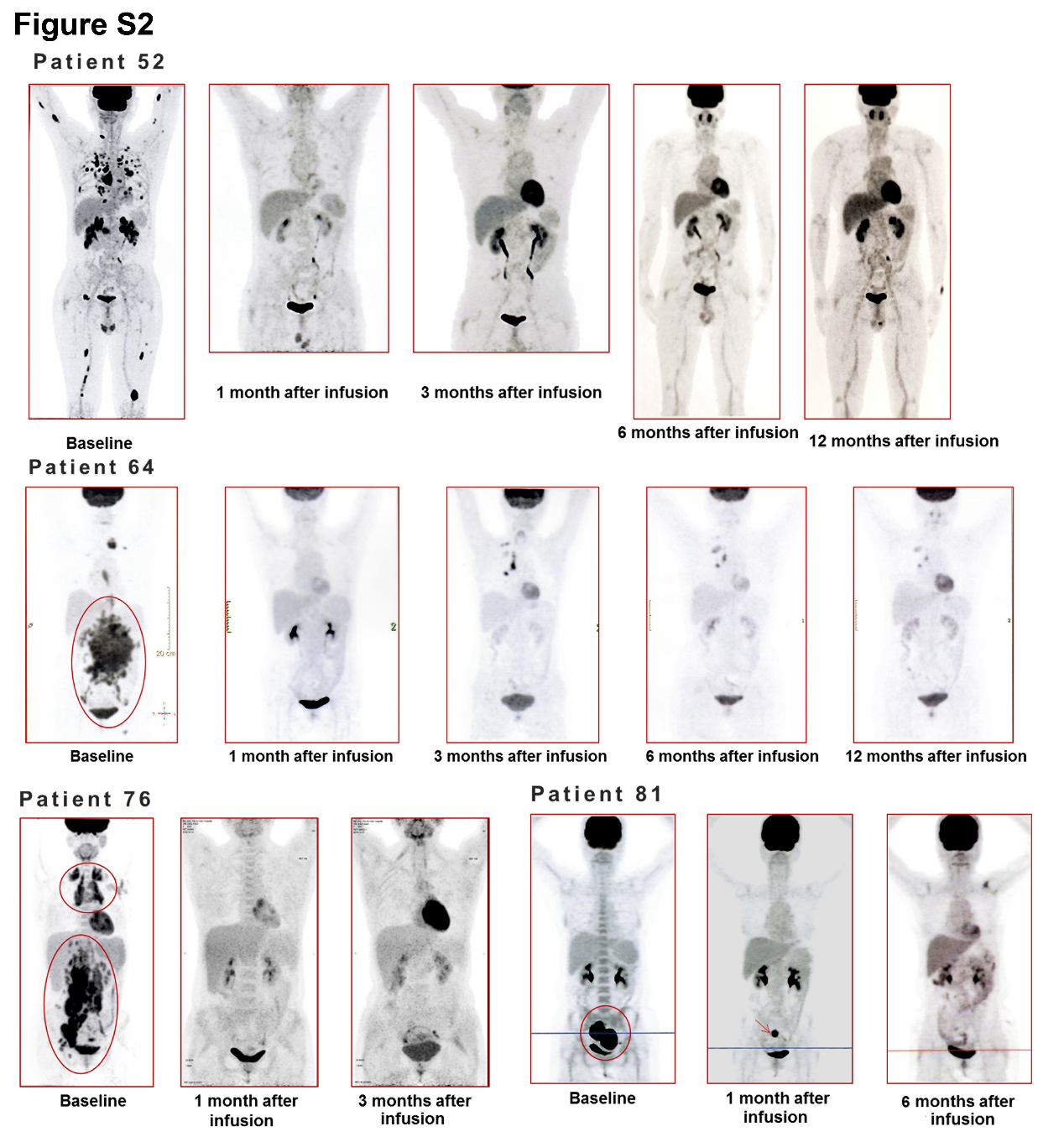


**Figure S3. Progression-free survival among patients with SPD ≥ 100 cm^2^**

SPD, sum of the product of the diameters.

**
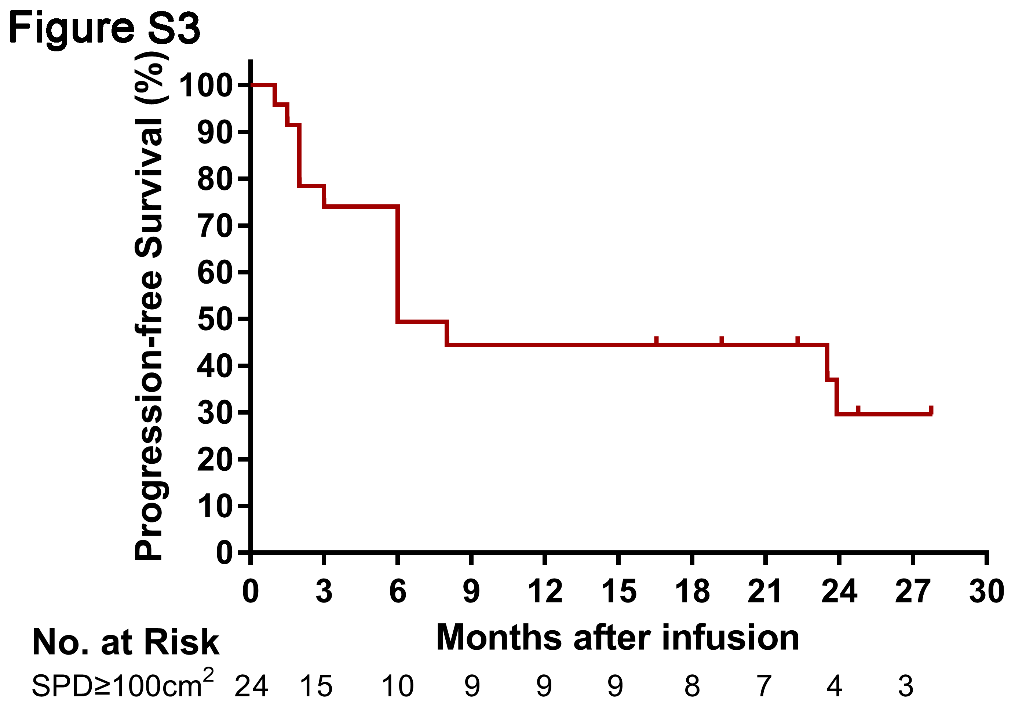
**

**Figure S4. Progression-free survival among patients with different dose of infusion**


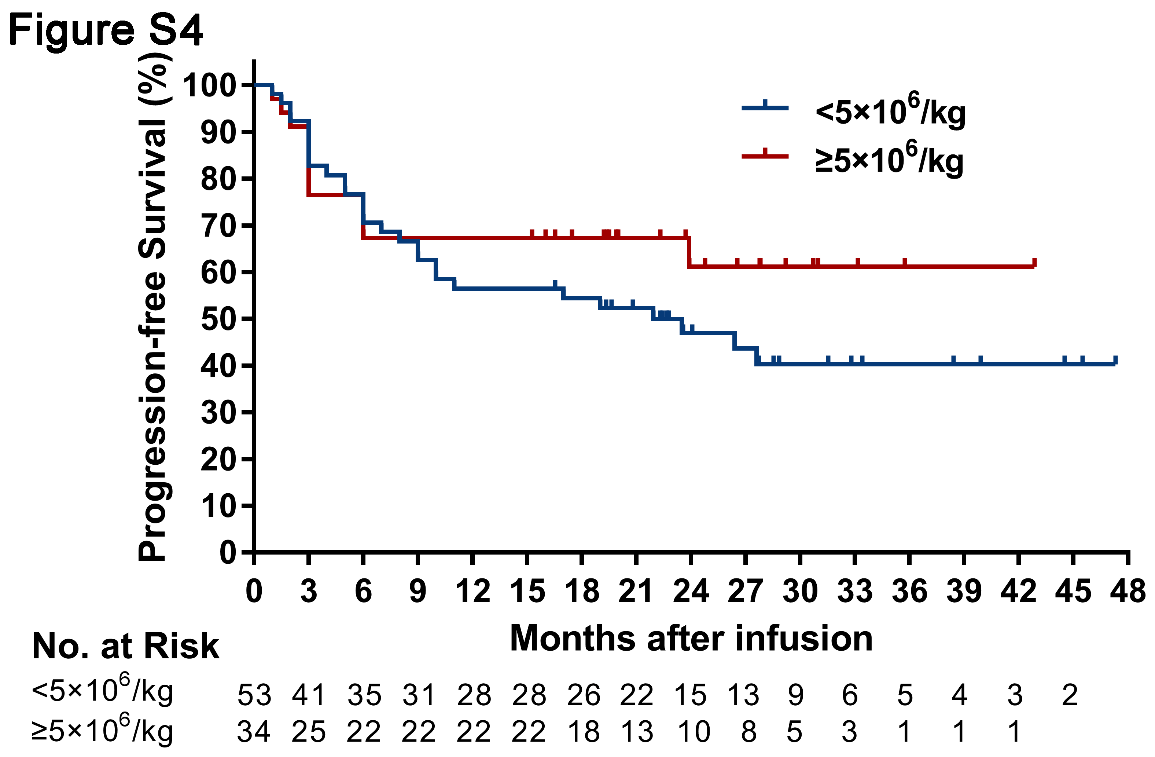


**Figure S5. Kaplan-Meier Estimates of the Duration of Response, Progression-free Survival, and Overall Survival among patients with DLBCL**

DLBCL, diffuse large B-cell lymphoma; GCB, germinal centre‐derived B-cell lymphoma; non-GCB, non-germinal centre‐derived B-cell lymphoma.

**
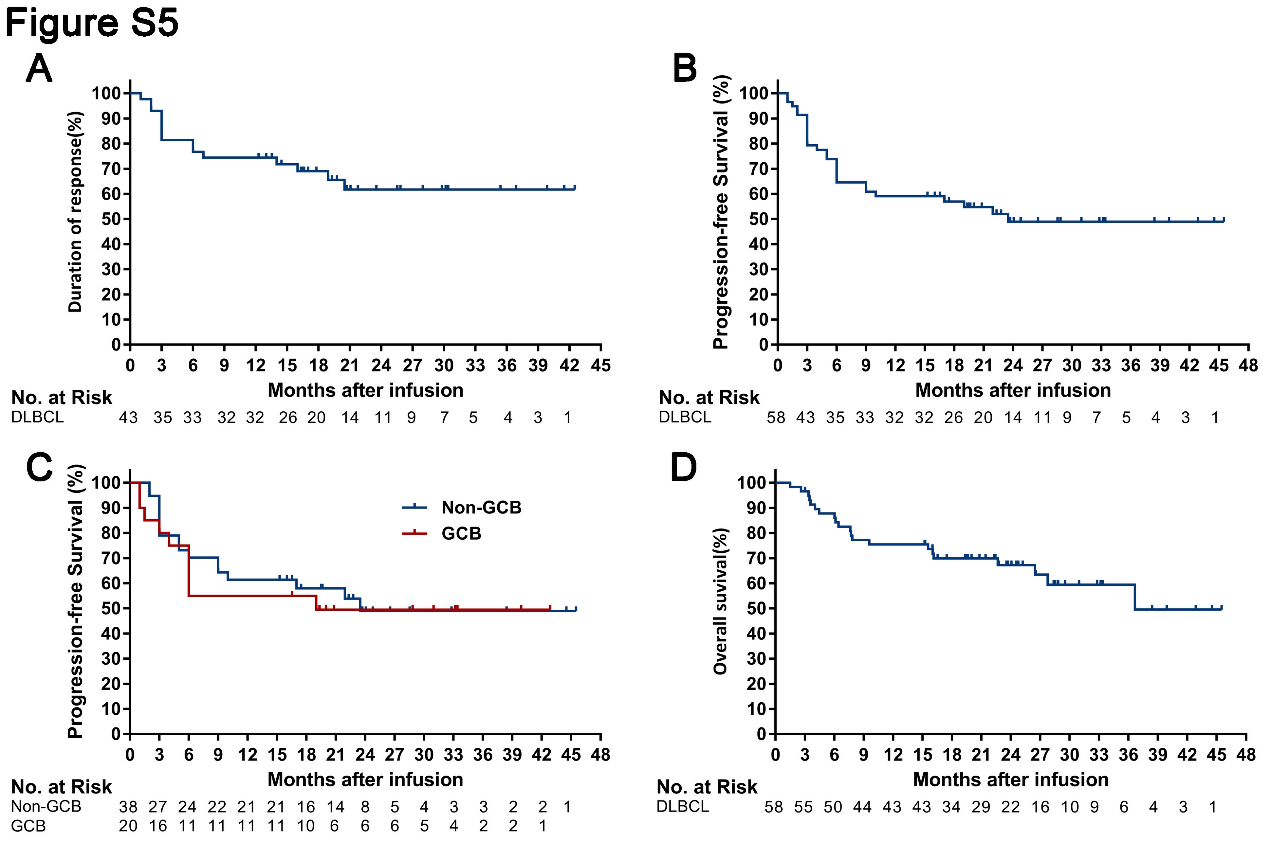
**

**Figure S6. Duration of response among patients who experienced prior CAR19 or no history of CAR19**

**
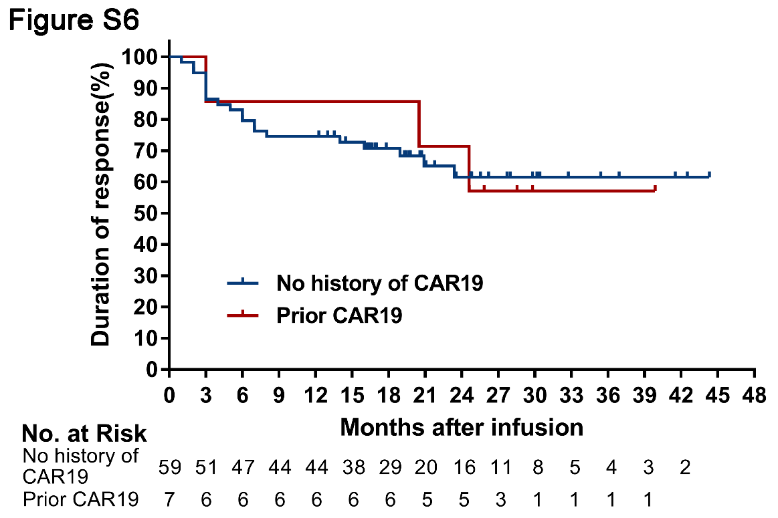
**

**Figure S7. The Correlations of CRS with infusion dose, tumour burden and expansion of CAR T cells**

The panel shows the associations of CRS with infusion dose (A), tumour burden (B, C) and expansion of CAR T cells (D). The greater the SPD was, the greater the probability of high-grade CRS (E); the stronger the CAR T amplification was, the greater the probability of CRS (F). P values were calculated by the Wilcoxon rank sum test. CAR, chimeric antigen receptor; CRS, cytokine release syndrome; GTD, greatest tumour diameter; SPD, sum of the product of the diameters.

**
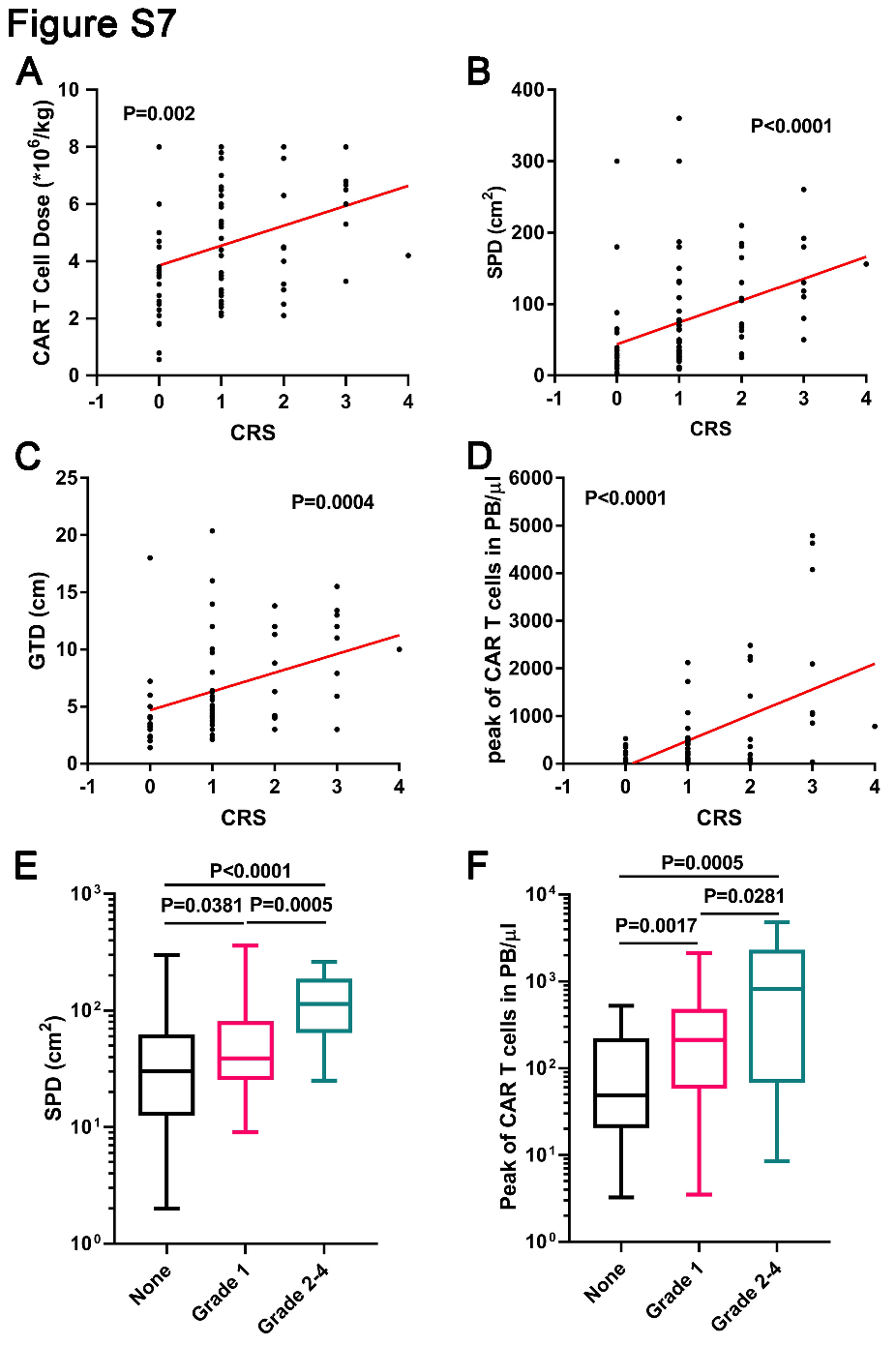
**

**Figure S8. Serum biomarkers associated with CRS**

The peak value is defined as the maximum level of the cytokine after baseline. Cytokine release syndrome was associated with the peak values of CRP, IL-2, IL-6 and TNF-α but not with those of IL-8 and IL-10. CRS, cytokine release syndrome.


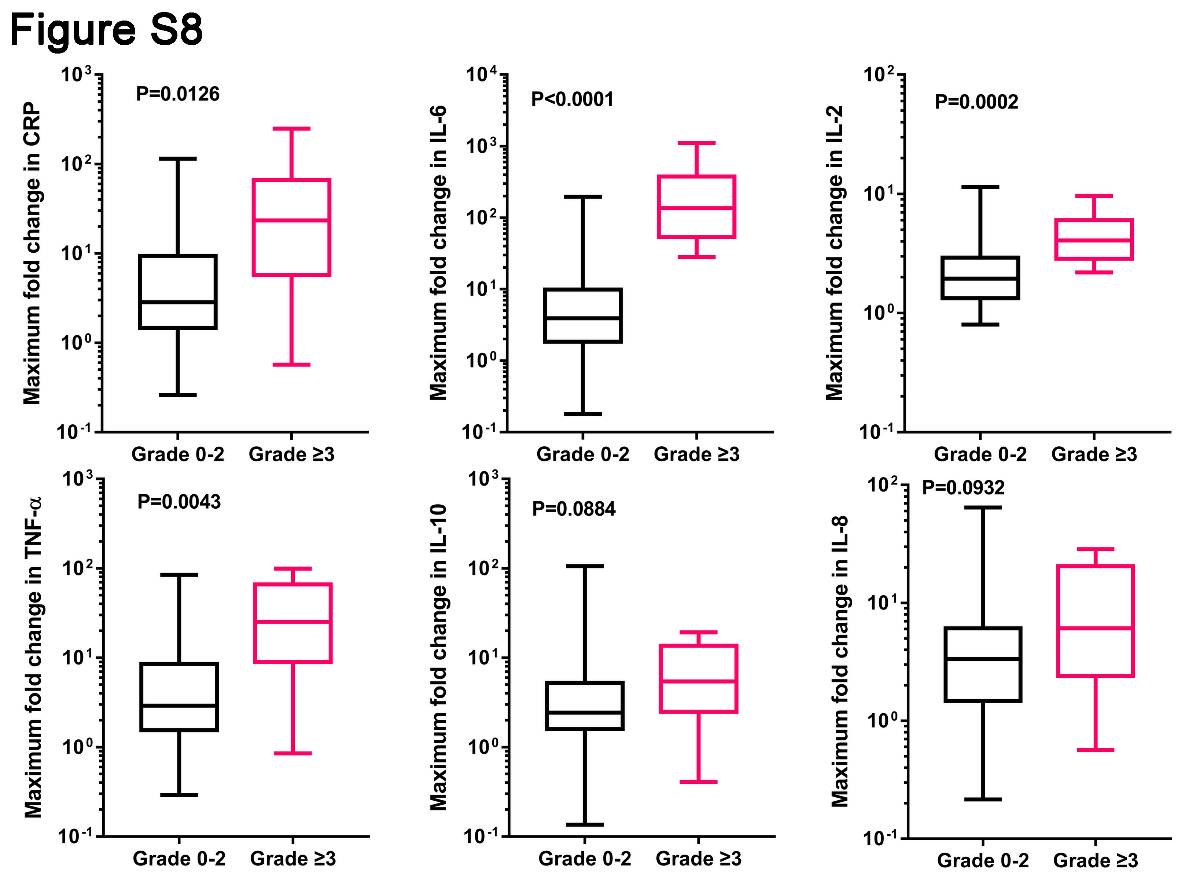


**Figure S9. The Correlations of CRES with infusion dose**

CRES, CAR T cell-related encephalopathy syndrome.

**
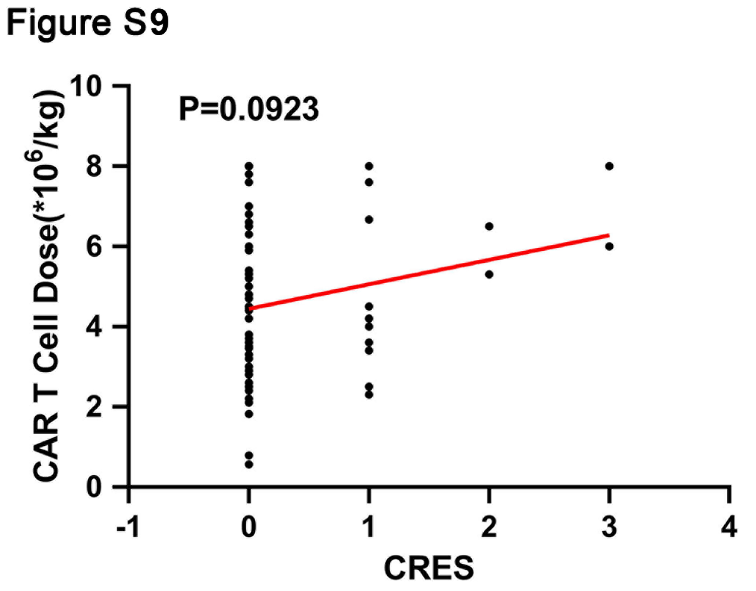
**

**Figure S10 Bone marrow suppression and recovery**

FC, cyclophosphamide and fludarabine; FC+D, cyclophosphamide, fludarabine and doxorubicin; WBC, white blood cell.


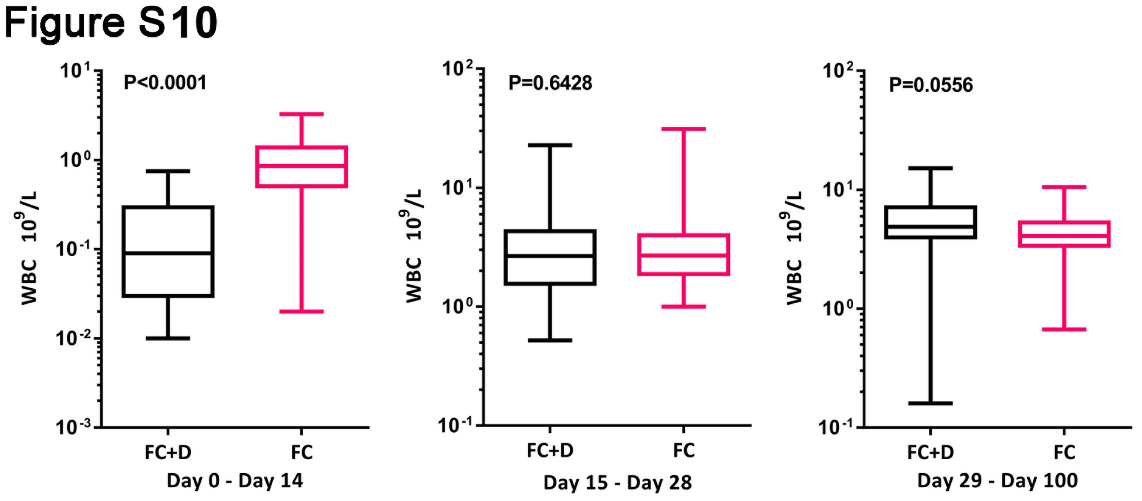


**Figure S11. B-cell concentrations**

All patients who had a response and had assessable samples were included in this analysis. The time of the day before conditioning regimen was used as the origin point. B cell recovery was defined as reaching ≥1% CD19+ cells in viable white blood cells or ≥3% CD19+ cells in lymphocytes in blood.

**
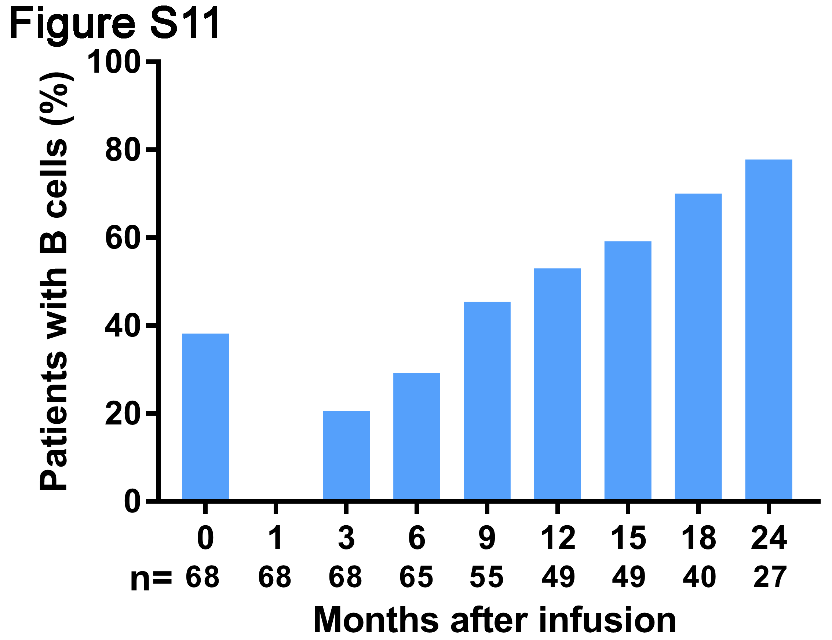
**

**Figure S12. CAR T cell expansion and correlations with response**

The in vivo peak expansion and persistence of TanCAR7 T cells in the peripheral blood of patients were analysed according to the percent of CAR T cells in T cells (A), absolute number of CAR T cells per microlitre of blood (B), and the copy number of the TanCAR7 transgene (C). The association between CAR T cell expansion and response was measured as the peak percent of CAR T cells in T cells, levels of CAR cells per microlitre of blood (D), or area under the curve (E), with the objective response (Response and non-Response). The CAR AUC was defined as the cumulative levels of CAR-positive cells/μL of blood over the first 28 days post TanCAR7 T cell infusion. The association between CAR T cell persistence and relapse was measured as the blood CAR T cell levels at 21-40 and 41-60 days between patients who had an ongoing response and those who had relapsed at the time of the data cut-off (F). AUC, area under the curve.


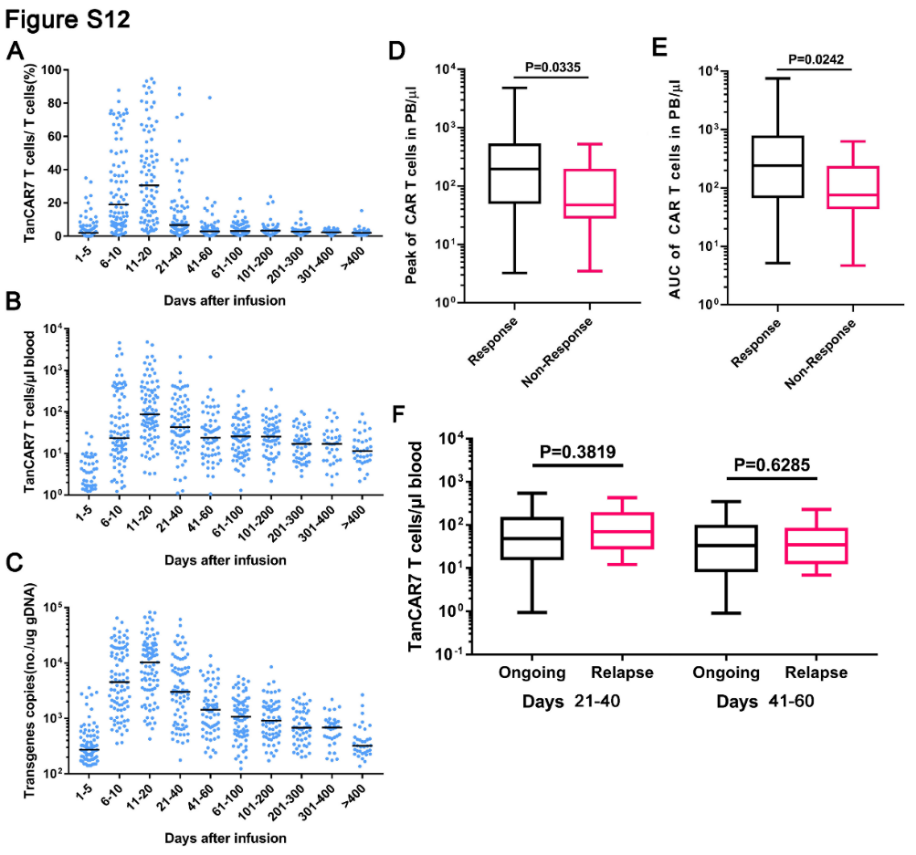


**Table S1. Demographic and clinical characteristics of the patients at baseline (Extended population) ^$^**

| Patient No. | Age (years) | Sex | Lymphoma Type | Disease stage at study entry | Lymphoma Status* | ECOG score | SPD (cm^2^) | CD19 (IHC) | CD20 (IHC) | BCL-2 (IHC) | BCL-6 (IHC) | c-Myc (IHC) | Ki-67 (IHC) |
| --- | --- | --- | --- | --- | --- | --- | --- | --- | --- | --- | --- | --- | --- |
| 30 | 61 | Male | DLBCL/non-GCB | IV | Refractory^#^ | 2 | 72 | + | +++ | + | + | 10% | 80% |
| 31 | 56 | Female | DLBCL/GCB | IV | Refractory^#^ | 2 | 28 | NA | + | + | + | 30% | 80% |
| 32 | 30 | Female | PMBCL/non-GCB | II | Refractory^#^ | 1 | 15 | + | +++ | + | + | 25% | 75% |
| 33 | 53 | Male | DLBCL/non-GCB | III | Refractory^#^ | 2 | 48 | + | + | + | + | 40% | 90% |
| 34 | 66 | Male | DLBCL/GCB | IV | Refractory | 2 | 63 | + | ++ | + | + | 41% | 91% |
| 35 | 67 | Female | DLBCL/non-GCB | IV | Refractory^#^ | 2 | 35 | NA | + | + | + | 40% | 90% |
| 36 | 34 | Male | DLBCL/GCB | IV | Refractory | 2 | 9 | NA | + | + | + | 30% | 75% |
| 37 | 60 | Male | DLBCL/non-GCB | IV | Refractory | 2 | 39 | + | + | + | + | 30% | 40% |
| 38 | 47 | Male | CLL/SLL | IV | Refractory | 2 | 80 | + | + | NA | NA | NA | 70% |
| 39 | 46 | Male | DLBCL/GCB | III | Relapsed | 1 | 2 | + | + | + | - | NA | 90% |
| 40 | 50 | Male | DLBCL/non-GCB | IV | Refractory^#^ | 1 | 10 | + | + | + | + | 60% | 50% |
| 41 | 46 | Female | FL | IV | Refractory^#^ | 2 | 46 | + | + | + | - | 10% | 60% |
| 42 | 53 | Male | DLBCL/GCB | IV | Refractory | 2 | 185 | + | + | + | + | - | 90% |
| 43 | 39 | Female | FL | IV | Refractory | 1 | 30 | + | + | + | + | NA | 40% |
| 44 | 40 | Female | DLBCL/GCB | IV | Refractory^#^ | 2 | 156 | + | + | + | + | 50% | 70% |
| 45 | 35 | Female | FL | IV | Refractory^#^ | 2 | 180 | + | +++ | + | + | NA | 10% |
| 46 | 45 | Female | FL | IV | Refractory^#^ | 2 | 130 | + | + | + | NA | NA | 40% |
| 47 | 51 | Male | TFL/non-GCB | IV | Refractory | 2 | 181 | + | + | ++ | + | 30% | 60% |
| 48 | 62 | Male | DLBCL/non-GCB | IV | Refractory^#^ | 2 | 35 | + | + | + | + | - | 80% |
| 49 | 48 | Male | DLBCL/non-GCB | IV | Refractory^#^ | 2 | 10 | + | + | + | + | + | 80% |
| 50 | 39 | Female | DLBCL/GCB | III | Relapsed | 1 | 118 | + | + | + | + | 10% | 80% |
| 51 | 41 | Female | DLBCL/non-GCB | III | Refractory^#^ | 1 | 64 | + | ++ | + | - | 10% | 70% |
| 52 | 49 | Male | DLBCL | IV | Refractory^#^ | 0 | 105 | + | + | + | + | 10% | 70% |
| 53 | 38 | Male | DLBCL/non-GCB | IV | Refractory^#^ | 1 | 109 | + | ++ | + | - | 40% | 90% |
| 54 | 31 | Male | DLBCL/non-GCB | III | Refractory^#^ | 1 | 70 | + | +++ | + | - | 40% | 100% |
| 55 | 66 | Female | DLBCL/non-GCB | III | Refractory^#^ | 0 | 10 | + | + | + | + | 40% | 70% |
| 56 | 54 | Female | DLBCL/non-GCB | III | Refractory^#^ | 1 | 12.86 | + | + | + | 50% | 10% | 80% |
| 57 | 43 | Male | DLBCL/non-GCB | II | Refractory^#^ | 0 | 10.25 | ++ | + | + | + | 25% | 70% |
| 58 | 64 | Male | DLBCL/non-GCB | II | Refractory | 0 | 3.28 | + | + | 80% | + | 40% | 80% |
| 59 | 49 | Male | DLBCL/non-GCB | II | Refractory^#^ | 0 | 32.74 | + | + | 30% | + | 50% | 90% |
| 60 | 18 | Female | PMBCL/non-GCB | IV | Refractory^#^ | 0 | 54 | + | + | + | 30% | 30% | 25% |
| 61 | 50 | Female | DLBCL/non-GCB | III | Refractory | 0 | 25 | + | + | + | 20% | 60% | 50% |
| 62 | 55 | Male | TFL/non-GCB | IV | Refractory^#^ | 0 | 25.65 | + | + | + | - | - | 30% |
| 63 | 54 | Male | DLBCL/non-GCB | II | Refractory | 0 | 31.1 | + | +++ | + | + | 20% | 60% |
| 64 | 57 | Female | FL/GCB | III | Refractory^#^ | 1 | 180 | + | + | + | + | 5% | 10% |
| 65 | 45 | Male | DLBCL/non-GCB | III | Refractory^#^ | 0 | 25 | ++ | ++ | 75% | + | - | 75% |
| 66 | 60 | Female | FL/ GCB | IV | Refractory | 2 | 300 | + | + | + | + | - | 5% |
| 67 | 37 | Male | DLBCL/ GCB | II | Refractory^#^ | 0 | 22.5 | + | + | - | + | 40% | 90% |
| 68 | 32 | Female | PMBCL/non-GCB | IV | Refractory^#^ | 0 | 130 | + | + | + | + | + | 80% |
| 69 | 43 | Female | DLBCL/non-GCB | IV | Refractory^#^ | 1 | 187 | + | + | + | + | 40% | 80% |
| 70 | 37 | Male | DLBCL/ GCB | IV | Refractory^#^ | 1 | 90 | + | + | - | + | 60% | 95% |
| 71 | 46 | Female | MALT /non-GCB | IV | Relapsed | 0 | 75 | + | + | + | 20% | 20% | 20% |
| 72 | 52 | Female | DLBCL/non-GCB | IV | Refractory | 1 | 65 | + | +++ | + | - | 30% | 80% |
| 73 | 17 | Male | DLBCL/non-GCB | III | Refractory^#^ | 0 | 50 | + | + | + | + | 30% | 90% |
| 74 | 64 | Male | TFL/non-GCB | IV | Refractory | 1 | 30.3 | + | + | + | + | 10% | 90% |
| 75 | 28 | Female | DLBCL/ GCB | IV | Relapsed | 0 | 26.3 | + | + | + | + | 30% | 60% |
| 76 | 30 | Female | FL/ GCB | III | Refractory^#^ | 0 | 260.3 | + | + | + | + | 5% | 20% |
| 77 | 31 | Male | DLBCL/ GCB | II | Refractory | 0 | 35.42 | ++ | +++ | + | + | 40% | 90% |
| 78 | 53 | Male | DLBCL/non-GCB | III | Relapsed | 0 | 35 | + | + | 90% | + | 30% | 80% |
| 79 | 53 | Male | DLBCL/non-GCB | II | Refractory | 0 | 30 | + | + | 80% | + | 20% | 70% |
| 80 | 53 | Female | DLBCL/ GCB | III | Refractory^#^ | 0 | 70 | + | +++ | 90% | 40% | 5% | 70% |
| 81 | 58 | Female | DLBCL/non-GCB | III | Relapsed | 1 | 150 | + | + | 90% | 70% | 5% | 50% |
| 82 | 33 | Male | PMBCL/ GCB | IV | Refractory^#^ | 2 | 180 | + | + | + | + | 10% | 80% |
| 83 | 26 | Female | PMBCL/non-GCB | III | Refractory^#^ | 0 | 30 | + | + | + | - | 5% | 50% |
| 84 | 41 | Female | DLBCL/non-GCB | IV | Relapsed | 1 | 30 | + | ++ | + | - | 90% | 60% |
| 85 | 57 | Female | DLBCL/ GCB | IV | Relapsed | 2 | 165 | + | + | + | 40% | 40% | 50% |
| 86 | 57 | Female | DLBCL/non-GCB | IV | Relapsed | 2 | 25 | + | + | + | + | + | 40% |
| 87 | 33 | Female | DLBCL/ GCB | IV | Refractory^#^ | 1 | 30 | + | + | + | + | 90% | 60% |
| 88 | 41 | Male | DLBCL/non-GCB | III | Refractory^#^ | 1 | 108 | + | + | 90% | 50% | 10% | 50% |

^$^The baseline characteristics of the first 29 patients have been reported previously^6^.

*Refractory was defined as no response after the last line of chemotherapy or relapse within 1 year of ASCT. ^#^Primary refractory.

CLL/SLL, chronic lymphocytic leukaemia and small lymphocytic lymphoma; DLBCL, diffuse large B-cell lymphoma; ECOG, Eastern Cooperative Oncology Group; FL, follicular lymphoma; GCB, germinal centre‐derived B-cell lymphoma; IHC, immunohistochemistry; MALT, mucosa-associated lymphoid tissue lymphoma; MCL, mantle-cell lymphoma; non-GCB, non-germinal centre‐derived B-cell lymphoma; NA, not applicable; PMBCL, primary mediastinal B-cell lymphoma; SPD, sum of the product of the diameters; TFL, transformed follicular lymphoma.

**Table S2. Characteristics of infused CAR T cells (Extended population) ^$^**

| Patient No. | CAR T Cell Dose(*10^6^/kg) | % Transduced | %CD3^+^ | %CD3^+^CD4^+^ | %CD3^+^CD8^+^ | %CD3^+^CD62L^+^  CD45RO^+^ | % cell viability |
| --- | --- | --- | --- | --- | --- | --- | --- |
| 30 | 4 | 37.1 | 98.4 | 39.1 | 55.69 | 61.9 | 93 |
| 31 | 8 | 35.9 | 98.1 | 24.9 | 68.6 | 63 | 92 |
| 32 | 6 | 11.5 | 99.7 | 12.8 | 79.6 | 60.1 | 88 |
| 33 | 3 | 18.4 | 99.5 | 85.4 | 13.1 | 68.1 | 94 |
| 34 | 4.46 | 20.7 | 99.1 | 34.6 | 59.9 | 41.9 | 92 |
| 35 | 3.6 | 38.2 | 98 | 47.1 | 49.6 | 64.9 | 96 |
| 36 | 3 | 17.6 | 99.6 | 10.6 | 86.4 | 43.8 | 87 |
| 37 | 3 | 38.4 | 99.9 | 39.6 | 59.3 | 65.4 | 86 |
| 38 | 6 | 21.2 | 99.2 | 23.8 | 70.5 | 69.4 | 94 |
| 39 | 4.5 | 18 | 99 | 34.8 | 63.2 | 30.3 | 93 |
| 40 | 3.8 | 28.6 | 98.7 | 56.5 | 38.7 | 78.7 | 87 |
| 41 | 6 | 21.4 | 96.8 | 48.1 | 44.3 | 49 | 91 |
| 42 | 8 | 27.6 | 98.4 | 18.6 | 79.6 | 48.2 | 86 |
| 43 | 3.8 | 16.5 | 99.6 | 26.3 | 69.6 | 34.4 | 85 |
| 44 | 4.2 | 24.2 | 98.7 | 18.6 | 76.3 | 73.3 | 91 |
| 45 | 8 | 36.9 | 93.6 | 43.2 | 45.9 | 50.4 | 90 |
| 46 | 5.2 | 22.2 | 98.6 | 21.5 | 73.1 | 85.5 | 94 |
| 47 | 4.5 | 18.3 | 93.6 | 33.6 | 56.7 | 58 | 94 |
| 48 | 3.5 | 24.2 | 94.4 | 57.4 | 31.4 | 74.1 | 86 |
| 49 | 5.9 | 25.4 | 95.9 | 16 | 73.4 | 15.9 | 94 |
| 50 | 3.3 | 28.2 | 98 | 46.4 | 47.2 | 24.8 | 91 |
| 51 | 2.5 | 21.5 | 91.7 | 10.1 | 79.5 | 38.9 | 86 |
| 52 | 8 | 16.3 | 97.5 | 11.4 | 80.5 | 13.8 | 87 |
| 53 | 2.8 | 16 | 97.2 | 14.4 | 81.3 | 19.4 | 93 |
| 54 | 7.6 | 19.9 | 97.7 | 51.8 | 46.5 | 14.8 | 95 |
| 55 | 2.1 | 25.4 | 97.6 | 47.4 | 46.4 | 55.9 | 82 |
| 56 | 8 | 25.5 | 94.5 | 44 | 48.8 | 39.3 | 80 |
| 57 | 8 | 23.8 | 94.7 | 24 | 70.1 | 17 | 88 |
| 58 | 3.2 | 17.9 | 98.1 | 24.6 | 70.9 | 26.3 | 92 |
| 59 | 3 | 23.6 | 97.4 | 53.1 | 43.2 | 20.8 | 92 |
| 60 | 3.2 | 29.9 | 97.8 | 47.3 | 49.4 | 25.7 | 91 |
| 61 | 2.9 | 30.2 | 91.4 | 39.4 | 52.3 | 56.9 | 80 |
| 62 | 3.6 | 18.8 | 98.4 | 64.2 | 27.4 | 74.4 | 87 |
| 63 | 2.6 | 11.7 | 95.5 | 30.1 | 61.1 | 58.9 | 90 |
| 64 | 8 | 36.4 | 96.2 | 45.9 | 50.1 | 34.8 | 94 |
| 65 | 2.2 | 25.8 | 99.3 | 30.9 | 61.1 | 7.7 | 93 |
| 66 | 3.5 | 35.5 | 95.3 | 19.1 | 69.3 | 74 | 85 |
| 67 | 2.1 | 26.7 | 96.8 | 20.9 | 75.3 | 16.1 | 94 |
| 68 | 3 | 14.4 | 99.6 | 19.1 | 73.4 | 7.2 | 92 |
| 69 | 6 | 20.5 | 99.7 | 31.4 | 59.7 | 30.2 | 91 |
| 70 | 7 | 21.7 | 92.2 | 28.9 | 50.8 | 37.8 | 89 |
| 71 | 6.5 | 29.1 | 97.6 | 6 | 89.2 | 57.6 | 83 |
| 72 | 4.4 | 16.7 | 99.6 | 45.8 | 48.1 | 28.9 | 85 |
| 73 | 6 | 17.1 | 99.9 | 15.9 | 78.8 | 85.4 | 92 |
| 74 | 6.6 | 19 | 99.7 | 38.5 | 53.4 | 86 | 90 |
| 75 | 3.5 | 17.6 | 97.6 | 41.9 | 56.3 | 46.7 | 88 |
| 76 | 8 | 22.6 | 92.8 | 45.4 | 45.8 | 28.8 | 83 |
| 77 | 2.6 | 33.2 | 98.6 | 61.3 | 36.3 | 32.6 | 92 |
| 78 | 6.5 | 15.5 | 93.2 | 39.4 | 50.4 | 90.7 | 89 |
| 79 | 4.2 | 10.1 | 98.5 | 39.8 | 56.9 | 49 | 84 |
| 80 | 4.8 | 11.5 | 97.7 | 15.5 | 83 | 70 | 94 |
| 81 | 6.3 | 14.5 | 95.1 | 38.1 | 55.2 | 59.5 | 92 |
| 82 | 3.8 | 11.1 | 98.7 | 17.7 | 79.6 | 51.5 | 92 |
| 83 | 8 | 16.8 | 98.8 | 23.4 | 74.5 | 19.1 | 86 |
| 84 | 8 | 12.7 | 94.8 | 20.5 | 70.9 | 49.1 | 85 |
| 85 | 7.6 | 27.1 | 95.9 | 27.7 | 69.6 | 68.2 | 81 |
| 86 | 6.3 | 10.5 | 98.8 | 30.8 | 64.9 | 42.4 | 91 |
| 87 | 4.5 | 18.3 | 96.8 | 34.4 | 60.2 | 34.8 | 90 |
| 88 | 2.1 | 12.9 | 96.7 | 57 | 37.5 | 48.1 | 90 |

^$^The characteristics of the infused cells have been reported previously^6^.

**Table S3. Baseline characteristics of relapsed patients**

| **Subgroup** | **No. of complete remission** | **No. of relapsed** |
| --- | --- | --- |
| **All** | 61 | 16 |
| **ECOG performance-status score-no. (%)** |  |  |
| 0-1 | 41 | 8 |
| 2 | 20 | 8 |
| **Disease stage at study entry** |  |  |
| I or II | 9 | 0 |
| III or IV | 52 | 16 |
| **Diagnosis on central histologic review-no. (%)** |  |  |
| DLBCL | 40 | 12 |
| TFL | 10 | 1 |
| FL | 5 | 2 |
| PMBCL | 2 | 0 |
| Others | 4 | 1 |
| **Double or triple expressor: MYC plus BCL2,BCL6 or both-no. (%)** |  |  |
| Yes | 19 | 6 |
| No | 26 | 5 |
| Miss data | 16 | 5 |
| **No. of previous lines of anti-neoplastic therapy-no. (%)** |  |  |
| ≤2 | 15 | 3 |
| 3-5 | 39 | 12 |
| ≥6 | 7 | 1 |
| **Tumor burden** |  |  |
| SPD ≥100cm^2^ | 14 | 7 |
| SPD <100cm^2^ | 47 | 9 |
| **Extranodal disease** |  |  |
| Yes | 39 | 12 |
| No | 22 | 4 |

DLBCL, diffuse large B-cell lymphoma; ECOG, Eastern Cooperative Oncology Group; FL, follicular lymphoma; PMBCL, primary mediastinal B-cell lymphoma; SPD, sum of the product of the diameters; TFL, transformed follicular lymphoma.

**Table S4. Treatment-associated adverse events in all treated patients within one month post infusion**

| Symptoms | G1 | G2 | G3 | G4 | Total |
| --- | --- | --- | --- | --- | --- |
| Any | 2 | 15 | 22 | 48 | 87 |
| Leukopenia | 4 | 11 | 20 | 46 | 81 |
| Pyrexia | 24 | 27 | 29 | 0 | 80 |
| Anorexia | 56 | 7 | 1 | 0 | 64 |
| Cytokine released syndrome | 39 | 13 | 8 | 1 | 61 |
| Fatigue | 45 | 11 | 4 | 0 | 60 |
| Triglyceride increased | 51 | 4 | 4 | 0 | 59 |
| Thrombocytopenia | 18 | 6 | 18 | 14 | 56 |
| Anemia | 17 | 18 | 14 | 3 | 52 |
| Serum ferritin increased | 30 | 17 | 2 | 0 | 49 |
| Herpes zoster | 29 | 4 | 1 | 0 | 34 |
| Tachycardia | 26 | 7 | 1 | 0 | 34 |
| Aspartic transaminase increased | 20 | 10 | 2 | 0 | 32 |
| Gamma-glutamyltransferase increased | 20 | 9 | 1 | 0 | 30 |
| Alanine aminotransferase increased | 20 | 9 | 1 | 0 | 30 |
| Cough | 23 | 3 | 1 | 0 | 27 |
| Rash | 25 | 2 | 0 | 0 | 27 |
| Loss of weight | 22 | 4 | 0 | 0 | 26 |
| Arthralgia | 21 | 2 | 1 | 0 | 24 |
| Hypokalemia | 22 | 2 | 0 | 0 | 24 |
| Hypoproteinemia | 22 | 1 | 0 | 0 | 23 |
| Hyponatremia | 17 | 2 | 1 | 1 | 21 |
| Nausea | 18 | 2 | 1 | 0 | 21 |
| Chill | 17 | 3 | 0 | 0 | 20 |
| Dizziness | 17 | 2 | 0 | 0 | 19 |
| Peripheral edema | 13 | 5 | 1 | 0 | 19 |
| Hypotension | 6 | 8 | 4 | 0 | 18 |
| Bacterial infection | 11 | 2 | 2 | 1 | 16 |
| Hypocalcemia | 9 | 6 | 1 | 0 | 16 |
| Headache | 13 | 2 | 0 | 0 | 15 |
| Vomiting | 10 | 2 | 1 | 0 | 13 |
| Diarrhea | 13 | 0 | 0 | 0 | 13 |
| Dental ulcer | 11 | 1 | 0 | 0 | 12 |
| Hyperglycemia | 12 | 0 | 0 | 0 | 12 |
| Hypophosphatemia | 10 | 0 | 0 | 0 | 10 |
| Tremor | 8 | 0 | 0 | 0 | 8 |
| Anxiety | 5 | 2 | 0 | 0 | 7 |
| Cavity effusion | 5 | 1 | 1 | 0 | 7 |
| Dyspnea | 1 | 1 | 3 | 1 | 6 |
| Total bilirubin increased | 4 | 1 | 1 | 0 | 6 |
| Constipation | 5 | 0 | 0 | 0 | 5 |
| Hypertension | 3 | 1 | 0 | 0 | 4 |
| Creatinine increased | 3 | 0 | 0 | 0 | 3 |
| Disturbance in attention | 2 | 1 | 0 | 0 | 3 |
| Leukocytosis | 2 | 0 | 0 | 0 | 2 |
| Seizure | 2 | 0 | 0 | 0 | 2 |
| Hyponoia | 0 | 1 | 0 | 0 | 1 |
| Dysgraphia | 0 | 1 | 0 | 0 | 1 |
| Disorientation | 0 | 1 | 0 | 0 | 1 |
| Confusion | 0 | 1 | 0 | 0 | 1 |
| Somnolence | 1 | 0 | 0 | 0 | 1 |
| Gatism | 1 | 0 | 0 | 0 | 1 |
| [Aphasis](file:///C:\youdao\dict\Application\8.5.1.0\resultui\html\index.html#/javascript:;) | 0 | 0 | 0 | 0 | 0 |
| Hypomagnesemia | 0 | 0 | 0 | 0 | 0 |
| Aphasia | 0 | 0 | 0 | 0 | 0 |
| Myasthenia | 0 | 0 | 0 | 0 | 0 |
| Intracranial hypertension | 0 | 0 | 0 | 0 | 0 |
| Papilledema | 0 | 0 | 0 | 0 | 0 |
| Encephaledema | 0 | 0 | 0 | 0 | 0 |

Individual symptoms of adverse events graded according to the Common Terminology Criteria for Adverse Events (CTCAE), version 4.0 are shown.

G1, grade 1; G2, grade 2; G3, grade 3; G4, grade 4.

1. Kochenderfer JN, Dudley ME, Kassim SH, Somerville RP, Carpenter RO, Stetler-Stevenson M*, et al.* Chemotherapy-refractory diffuse large B-cell lymphoma and indolent B-cell malignancies can be effectively treated with autologous T cells expressing an anti-CD19 chimeric antigen receptor. *J Clin Oncol* 2015 Feb 20; **33**(6)**:** 540-549.

2. Neelapu SS, Locke FL, Bartlett NL, Lekakis LJ, Miklos DB, Jacobson CA*, et al.* Axicabtagene Ciloleucel CAR T-Cell Therapy in Refractory Large B-Cell Lymphoma. *N Engl J Med* 2017 Dec 28; **377**(26)**:** 2531-2544.

3. Schuster SJ, Bishop MR, Tam CS, Waller EK, Borchmann P, McGuirk JP*, et al.* Tisagenlecleucel in Adult Relapsed or Refractory Diffuse Large B-Cell Lymphoma. *N Engl J Med* 2019 Jan 3; **380**(1)**:** 45-56.

4. Locke FL, Ghobadi A, Jacobson CA, Miklos DB, Lekakis LJ, Oluwole OO*, et al.* Long-term safety and activity of axicabtagene ciloleucel in refractory large B-cell lymphoma (ZUMA-1): a single-arm, multicentre, phase 1-2 trial. *Lancet Oncol* 2019 Jan; **20**(1)**:** 31-42.

5. Schuster SJ, Svoboda J, Chong EA, Nasta SD, Mato AR, Anak O*, et al.* Chimeric Antigen Receptor T Cells in Refractory B-Cell Lymphomas. *N Engl J Med* 2017 Dec 28; **377**(26)**:** 2545-2554.

6. Tong C, Zhang Y, Liu Y, Ji X, Zhang W, Guo Y*, et al.* Optimized tandem CD19/CD20 CAR-engineered T cells in refractory/relapsed B-cell lymphoma. *Blood* 2020 Oct 1; **136**(14)**:** 1632-1644.
